# Low-Magnitude Mechanical Signals to Preserve Skeletal Health in Female Adolescents With Anorexia Nervosa: A Randomized Clinical Trial

**DOI:** 10.1001/jamanetworkopen.2024.41779

**Published:** 2024-10-31

**Authors:** Amy D. DiVasta, Catherine Stamoulis, Clinton T. Rubin, Jenny Sadler Gallagher, Douglas P. Kiel, Brian D. Snyder, Catherine M. Gordon

**Affiliations:** 1 Division of Adolescent Medicine, Boston Children’s Hospital, Boston, Massachusetts; 2 Harvard Medical School, Boston, Massachusetts; 3 Department of Biomedical Engineering, State University of New York at Stony Brook, Stony Brook; 4 Hinda and Arthur Marcus Institute for Aging Research, Hebrew SeniorLife, Boston, Massachusetts; 5 Department of Medicine, Beth Israel Deaconess Medical Center, Boston, Massachusetts; 6 Department of Orthopedic Surgery, Boston Children’s Hospital, Boston, Massachusetts; 7 Eunice Kennedy Shriver National Institute of Child Health and Human Development, Bethesda, Maryland

## Abstract

**Importance:**

Malnourished adolescents and young adults with anorexia nervosa (AN) are at high risk for skeletal deficits.

**Objective:**

To examine whether low-magnitude mechanical signals (LMMS) could preserve bone mineral density (BMD) throughout 6 months in adolescents and young adults with AN.

**Design, Setting, and Participants:**

This double-blind, sham-controlled randomized clinical trial, conducted in a hospital-based specialty clinic, assessed female adolescents and young women without medical comorbidity or medication use that would compromise bone health. A total of 837 female adolescents were screened from January 1, 2012, to December 31, 2019, of whom 317 met the study criteria. Data analysis was performed from 2020 to 2024.

**Intervention:**

Platform delivering low-magnitude mechanical signals (LMMS) (0.3 g at 32-37 Hz) or sham (ie, placebo) signals for 10 minutes daily for 6 months.

**Main Outcomes and Measures:**

The primary outcome was trabecular volumetric BMD (vBMD) as measured by peripheral quantitative computed tomography of the tibia at baseline and 6 months. Secondary outcomes included cortical vBMD, cross-sectional area (CSA), areal BMD and body composition measured by dual-energy x-ray absorptiometry, and serum bone turnover markers.

**Results:**

Forty female adolescents and young women (median [IQR] age, 16.3 [15.1-17.6] years; median [IQR] percentage median BMI for age, 87.2% [81.0%-91.6%]) completed the trial. Total bone vBMD changes were nonsignificant in both groups (95% CI for difference in median change between groups, −57.11 to 2.49): in the LMMS group, vBMD decreased from a median (IQR) of 313.4 (292.9-344.6) to 309.4 (290.4-334.0) mg/cm^3^, and in the placebo group, it increased from a median (IQR) of 308.5 (276.7-348.0) to 319.2 (309.9-338.4) mg/cm^3^. Total CSA at the 4% tibia site increased from a median (IQR) of 795.8 (695.0-844.8) mm^2^ to 827.5 (803.0-839.4) mm^2^ in the LMMS group, whereas in the placebo group, it decreased from 847.3 (770.5-915.3) mm^2^ to 843.3 (828.9-857.7) mm^2^ (95% CI for difference in median change between groups, 2.94-162.53). Median (IQR) trabecular CSA at the 4% tibia site increased from 616.3 (534.8-672.3) mm^2^ to 649.2 (638.0-661.4) mm^2^ in the LMMS group but decreased in the placebo group from 686.4 (589.0-740.0) mm^2^ to 647.9 (637.3-661.9) mm^2^ (95% CI for difference in median change between groups, 2.80-139.68 mm^2^). Changes in cortical vBMD, cortical section modulus, and muscle CSA were not significant between groups. The 6-month changes in trabecular and total bone CSA at the tibia 4% site (weight-bearing trabecular bone) were significantly different between groups (these measures increased in the LMMS group but decreased in the placebo group; total bone CSA: 95% CI, 2.94-162.53; *P* = .01; trabecular CSA: 95% CI, 2.80-139.68; *P* = .02). Greater increases in body mass index were seen in the placebo group (median [IQR] gain, 0.5 [–0.3 to +2.1]) than in the LMMS group (median [IQR] gain, +0.4 [–0.3 to +2.1]), perhaps due to differences in fat mass accrual. No adverse events occurred related to the LMMS intervention.

**Conclusions and Relevance:**

In this randomized clinical trial of female adolescents and young women with AN, a 6-month LMMS intervention did not yield improvement in tibial trabecular vBMD. However, LMMS led to increases in total and trabecular CSA at the tibia. These results suggest an early positive response of increased bone turnover and trabecular bone quantity due to the LMMS intervention. Future studies should use a longer duration of intervention, consider strategies to optimize adherence, and potentially focus on a more profoundly malnourished patient population.

**Trial Registration:**

ClinicalTrials.gov Identifier: NCT01100567

## Key Points

**Question:**

Do low-magnitude mechanical signals (LMMS) improve bone health in adolescents with anorexia nervosa?

**Findings:**

In this randomized clinical trial of 50 female adolescents and young women, there was no difference in tibial trabecular volumetric bone mineral density (the study’s primary outcome). Tibial cross-sectional area increased in response to LMMS treatment but not in the sham group.

**Meaning:**

The findings suggest that an LMMS intervention may be a safe alternative to exercise for prevention of bone loss while patients are working toward weight restoration.

## Teaser

This randomized clinical trial of female adolescents with anorexia nervosa examines whether low-magnitude mechanical signals could preserve bone mineral density.

## Introduction

Increased fracture risk and early osteoporosis are known complications of anorexia nervosa (AN).^1,2,3^ Mechanisms of bone loss in affected adolescents include hormonal milieu changes, loss of lean body mass, and restriction of weight-bearing activity. Effective therapies for preventing bone loss in AN remain elusive. Hormonal therapies have yielded disappointing results and are associated with poor patient acceptance.^4^ As adolescence is a critical period for bone acquisition, identifying strategies to preserve bone mineral density (BMD) in adolescents with AN is paramount.

Physical activity increases BMD, yet clinicians avoid exercise in AN because it may interfere with weight restoration or cardiovascular safety. Low-magnitude mechanical signal (LMMS) stimulation may represent a nonpharmacologic intervention providing a weight-bearing stimulus to normalize skeletal health without requiring strenuous exertion that could compromise AN treatment goals.^5,6,7,8^ Extremely low-level (<100 microstrain), high-frequency (10-90 Hz) strains are anabolic to bone tissue.^6,9^ Brief, daily LMMS, delivered via a vibrating platform, can stimulate biomarkers of bone formation and preserve BMD in at-risk populations.^10,11,12^ We previously found that LMMS normalized bone turnover over 5 days in hospitalized patients with AN.^13^ We aimed to determine whether LMMS would affect BMD in ambulatory female adolescents with AN over 6 months. We hypothesized that LMMS would be anabolic to bone and that volumetric BMD would be greater in adolescents randomized to LMMS treatment compared with placebo treatment.

## Methods

### Patient Cohort

We screened 837 female adolescents and young women seen at Boston Children’s Hospital (BCH) from January 1, 2012, to December 31, 2019, of whom 317 met study criteria (Figure). Eligible patients were aged 11 to 26 years, had a body mass index (BMI) less than 20.0 (calculated as weight in kilograms divided by height in meters squared), had amenorrhea for more than 3 months, and exhibited disordered eating, distorted body image, and fear of weight gain consistent with AN diagnosis (*Diagnostic and Statistical Manual of Mental Disorders, Fifth Edition* [*DSM-5*]). Given the known association of race and ethnicity with BMD, participants were asked to self-identify their race and ethnicity on a demographic survey administered at the baseline study visit. Patients were excluded for other medical diagnoses or medication use that affected BMD. All patients provided written informed consent. The BCH Institutional Review Board approved the study. This report follows the Consolidated Standards of Reporting Trials (CONSORT) reporting guideline for randomized studies.^14^ The trial protocol can be found in Supplement 1.

**Figure.**
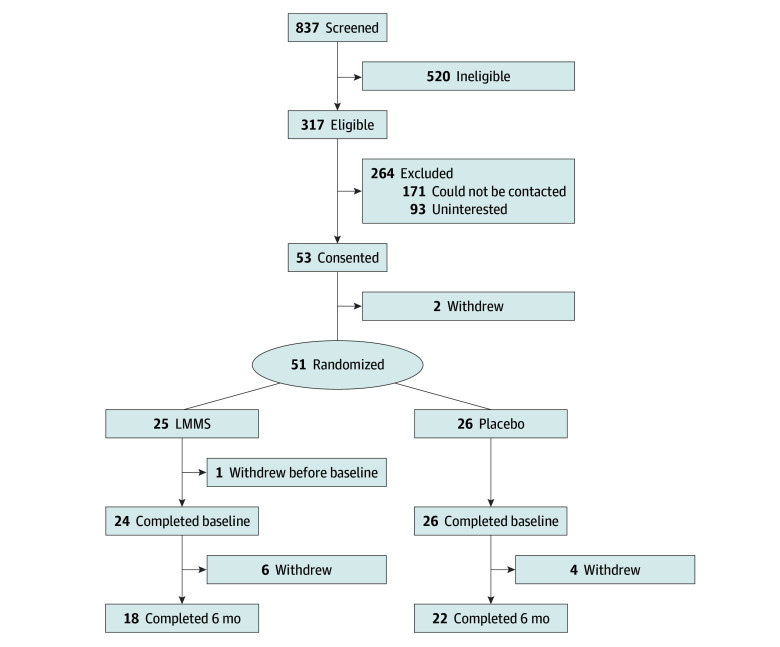
Participant Recruitment, Enrollment, and Disposition LMMS indicates low-magnitude mechanical signals.

### Study Design and Treatment

In this single-site, double-blind, placebo-controlled randomized clinical trial, 51 participants were include in intention-to-treat analysis (Figure). Treatment allocation (LMMS platform or sham platform) was randomly assigned using a randomly generated number list. Participants and investigators aside from the study coordinator and statistician were masked until trial completion. The treatment arm (n = 25) received a platform delivering LMMS using low-intensity vibration (0.3 g at 30 Hz ± 10%), a dose chosen to maximize safety and efficacy.^9,10,11^ The sham (placebo) arm (n = 26) received a platform identical in appearance and noise production to active platform but without vibration. A sample size of 25 participants per arm had 80% power to detect a 0.5% to 1.9% difference in mean realized gain between the arms.

Participants were instructed to stand on their platform 10 minutes daily for 6 months. Platform monitors tracked adherence electronically and prevented device overuse. Adherence was measured as platform-recorded minutes used every 3 months and as the ratio of minutes used to expected use (910 minutes at 3 months and 1820 minutes at 6 months). Study visits occurred in the BCH Experimental Therapeutics Unit at baseline, 3 months, and 6 months. Participants were advised to consume 1300 mg of calcium and 600 IU of vitamin D daily.

### Skeletal Assessments

Dual-energy x-ray absorptiometry (DXA) measurements of areal BMD were obtained for the total body less head, lumbar spine (L1-L4), and total hip and were analyzed using pediatric software.^15^ Body composition (total lean mass, total fat mass, and percentage of body fat) was also measured by DXA (Hologic Inc). Peripheral quantitative computed tomography measurements (pQCT) of left tibial cross-sections were obtained (Stratec XCT 3000, Orthometrix) at tibial length percentages proximal to the growth plate (4% and 66%). Our primary outcome was trabecular volumetric BMD (vBMD). Secondary outcomes included cortical vBMD, cross-sectional area (CSA), cortical thickness, cortical section modulus, and muscle CSA.

### Additional Study Assessments

Height and weight were measured, and percentage median BMI (%mBMI) was determined using the following formula: %mBMI = 100 × [Participant BMI/Median BMI for Age]. We obtained information about medications, healthy history, and family history. Fasting blood samples were collected every 3 months for the following biomarkers of bone formation: osteocalcin (enzyme-linked immunosorbent assay; coefficient of variation [CV], 5.0%-6.5%), bone-specific alkaline phosphatase (BSAP; competitive inhibition enzyme-linked immunosorbent assay [CIA]; CV, 1.5%-2.6%), and bone resorption (C-telopeptides; radioimmunoassay; CV, 5.2%-6.8%). Samples were also collected every 3 months for the following hormonal concentrations: adiponectin (enzyme immunoassay; CV, 5.0%-5.4%), leptin (radioimmunoassay; CV, 5.2%-7.5%), insulin (CIA; CV, 2.0%-4.2%), cortisol (CIA; CV, 4.4%-6.7%), and insulin-like growth factor 1 (IGF-1; EIA; CV, 6.6%-9.7%). Serum 25-hydroxyvitamin D (25[OH]D) (CIA; CV, 7.3%) and parathyroid hormone (CIA; CV, 5.4%-7.0%) were measured at baseline.

### Harmonization of Laboratory Data

During the trial, the BCH Experimental Therapeutics Unit switched from one research laboratory to another, causing some participants to have biomarker data collection performed by 2 laboratories (6 at baseline, 8 at 3 months, and 10 at 6 months). An analysis was conducted to assess processing-related differences introduced by the change and to harmonize the data. Models leveraged these data to estimate the relationship between the 2 sets of measurements; biomarker values were then estimated for those missing data in the set provided by the original laboratory. The maximum number of samples was 150 (50 participants measured at 3 points). Following this process, participants had complete biomarker data at baseline. Fifteen participants were missing these data at 6 months because they never provided blood samples.

### Statistical Analysis

Most analyzed variables were nonnormally distributed. Thus, summary statistics were reported as medians (IQRs). Statistical comparisons were performed using mixed-effects linear and quantile regression models (for comparison of results based on models with different statistical assumptions) to account for repeated measures for each participant (through a random intercept and slope) and assess change in the measures of interest from baseline to 6 months, within and across participants in each group. Primary models comparing baseline and 3- or 6-month outcomes included adjustments for age, AN duration, and weight gain. Additional models included additional parameters, such as duration of amenorrhea, %mBMI at baseline, treatment adherence, and/or baseline 25(OH)D concentration. Model adjustments are indicated in Table 1, Table 2, Table 3, and Table 4. Participants with %mBMI of 85% or less and those with %mBMI of 80% or less were separately examined. Additional comparisons of baseline data were performed using nonparametric Wilcoxon rank sum and signed-rank tests. Corresponding 95% CIs were estimated with bootstrapping (2000 draws). The significance level was set at α = .05. All analyses were conducted using Matlab, release R2023a (Mathworks Inc). Data analysis was performed from 2020 to 2024.

**Table 1.** Demographic and Clinical Characteristics at Baseline for All Participants and Those Randomized to LMMS or Placebo

Characteristic	All participants (N = 50), median (IQR) [range]	Median (IQR)		*P* value	95% CI for difference in group medians
		LMMS arm (n = 24)	Placebo arm (n = 26)		
Age, y	16.3 (15.1 to 17.6) [11.2 to 26.1]	16.9 (14.7 to 18.8)	16.3 (15.2 to 17.2)	.41	−0.68 to 1.86
Height, cm	161.7 (157.5 to 166.7) [139.3 to 181.9]	161.3 (156.6 to 164.2)	162.1 (157.8 to 166.8)	.52	−4.30 to 2.40
Weight, kg	45.6 (43.3 to 50.8) [29.5 to 60.9]	45.6 (39.1 to 50.7)	46.1 (44.1 to 50.9)	.44	−4.85 to 4.44
BMI	17.8 (16.4 to 18.7) [14.6 to 20.0]	17.4 (16.1 to 18.6)	18.2 (16.7 to 19.1)	.55	−1.90 to 0.60
Percentage of median BMI for age, %	87.2 (81.0 to 91.6) [51.2 to 102.1]	86.5 (80.1 to 92.0)	87.9 (83.7 to 91.6)	.42	−8.36 to 4.74
Duration of AN, mo	6.0 (3.0 to 12.0) [0 to 120.0]	6.5 (3.0 to 32.5)	6.0 (3.0 to 12.0)	.37	−4.0 to 18.50
Duration of amenorrhea, mo	6.0 (3.0 to 11.8) [0 to 78.0]	6.5 (3.0 to 15.0)	6.0 (2.0 to 11.0)	.68	−4.07 to 8.50
Age at menarche, y	12.0 (11.3 to 13.0) [10.0 to 15.0]	12.5 (12.0 to 13.0)	12.0 (11.0 to 13.0)	.41	−0.50 to 1.00
25(OH)D, ng/mL	36.9 (28.2 to 44.8) [18.3 to 68.1]	33.6 (23.1 to 42.3)	42.3 (33.3 to 49.6)	.04	−18.68 to −2.70
PTH, pg/mL	17.0 (10.8 to 21.3) [8.0 to 36.0]	17.0 (12.0 to 24.0)	18.0 (10.8 to 21.3)	.33	−6.50 to 6.00
Race and ethnicity, No. (%)					
Asian	3 (6.0)	2 (8.3)	1 (3.9)	.51	−11.73 to 22.28
Black	1 (2.0)	0	1 (3.9)	.33	−10.26 to 18.97
Hispanic	3 (6.0)	1 (4.2)	2 (7.7)	.61	−13.52 to 2.31
Non-Hispanic	47 (94.0)	23 (95.8)	24 (92.3)	.61	−13.52 to 2.31
White	44 (88.0)	20 (83.4)	24 (92.2)	.33	−10.28 to 28.95
Other^a^	2 (4.0)	2 (8.3)	0	.14	−5.90 to 25.81
Total body *z* score	−0.5 (−1.1 to 0.4) [−3.3 to 2.9]	−0.4 (−1.2 to 0.0)	−0.5 (−1.1 to 0.7)	.64	−1.15 to 0.60
Spine *z* score	−0.7 (−1.2 to 0.0) [−2.8 to 2.0]	−0.7 (−1.1 to −0.1)	−0.7 (−1.6 to 0.0)	.95	−0.60 to 0.75
Total hip *z* score	−0.4 (−1.1 to 0.1) [−3.1 to 3.0]	−0.6 (−1.1 to 0.0)	−0.4 (−1.1 to 0.4)	.34	−0.80 to 0.30
DXA total body *z* score, No. (%)					
≤–1	14 (28.0)	7 (29.2)	7 (28.0)	.93	−22.71 to 25.35
≤–2	3 (6.0)	2 (8.3)	1 (3.9)	.52	−11.82 to 22.20
DXA spine *z* score, No. (%)					
≤–1	18 (36.0)	9 (37.5)	9 (34.6)	.83	−22.28 to 27.85
≤–2	4 (8.0)	1 (4.2)	3 (11.5)	.35	−10.46 to 25.07
DXA total hip *z* score, No. (%)					
≤–1	15 (30.0)	7 (29.2)	8 (30.8)	.90	−22.97 to 25.53
≤–2	2 (4.0)	1 (4.2)	1 (3.9)	.96	−15.16 to 16.70

Abbreviations: 25(OH)D, 25-hydroxyvitamin D; AN, anorexia nervosa; BMI, body mass index (calculated as weight in kilograms divided by height in meters squared); DXA, dual energy x-ray absorptiometry; LMMS, low-magnitude mechanical signals; PTH, parathyroid hormone.

SI conversion factors: To convert 25(OH)D to nanomoles per liter, multiply by 2.4; PTH to nanograms per liter, multiply by 1.

^a^ No additional information available.

**Table 2.** Measures of Bone Density, Bone CSA, and Body Composition by Tibia Peripheral Quantitative Computed Tomography and DXA at Baseline and After 6 Months of LMMS Treatment or Placebo in 50 Young Women With Anorexia Nervosa

Measurement	Baseline median (IQR)	6-mo adjusted median (IQR)^a^	*P* value (within group, 0-6 mo)	95% CI for difference (0-6 mo) within group	*P* value (difference in change between groups)	95% CI for difference in median change between groups
**Tibia 4% site (weight-bearing trabecular bone)**						
Total bone vBMD, mg/cm^3^						
LMMS	313.4 (292.9 to 344.6)	309.4 (290.0 to 334.0)	.49	−28.21 to 18.45	.44	−57.11 to 2.49
Placebo	308.5 (276.7 to 348.0)	319.2 (309.9 to 338.4)	.45	−16.33 to 30.12		
Trabecular vBMD, mg/cm^3^						
LMMS	244.4 (224.8 to 273.6)	248.6 (233.1 to 257.2)	.86	−13.58 to 15.91	.84	−23.60 to 28.47
Placebo	250.0 (228.0 to 277.3)	249.8 (246.1 to 259.6)	.96	−18.75 to 18.28		
Total bone CSA, mm^2^						
LMMS	795.8 (695.0 to 844.8)	827.5 (803.0 to 839.4)	.33	−32.05 to 106.01	.01	2.94 to 162.53
Placebo	847.3 (770.5 to 915.3)	843.3 (828.9 to 857.7)	.43	−76.52 to 38.50		
Trabecular CSA, mm^2^						
LMMS	616.3 (534.8 to 672.3)	649.2 (638.0 to 661.4)	.11	−20.32 to 103.68	.02	2.80 to 139.68
Placebo	686.4 (589.0 to 740.0)	647.9 (637.3 to 661.9)	.30	−70.40 to 38.00		
Cortical vBMD, g/cm^3^						
LMMS	593.5 (505.1 to 629.4)	537.9 (508.8 to 603.8)	.16	−44.67 to 24.05	.07	−86.22 to 51.78
Placebo	551.2 (490.2 to 594.1)	563.2 (513.6 to 588.4)	.65	−32.79 to 68.63		
**Tibia 66% site (weight-bearing cortical bone)**						
Total bone vBMD, mg/cm^3^						
LMMS	599.7 (556.0 to 646.4)	626.9 (600.2 to 641.6)	.11	−18.99 to 75.13	.18	−35.80 to 96.08
Placebo	628.6 (570.1 to 681.1)	627.7 (610.9 to 645.7)	.95	37.29 to 43.51		
Cortical vBMD, g/cm^3^						
LMMS	1110.7 (1078.1 to 1118.5)	1100.3 (1086.9 to 1114.5)	.99	−14.68 to 9.41	.56	−19.21 to 6.00
Placebo	1089.3 (1073.1 to 1118.5)	1097.8 (1087.4 to 1101.6)	.34	−1.17 to 13.62		
Total bone CSA, mm^2^						
LMMS	503.5 (469.8 to 565.5)	508.6 (461.8 to 548.3)	.94	−34.79 to 49.02	.72	−30.20 to 50.88
Placebo	510.0 (477.5 to 553.0)	517.9 (490.2 to 547.6)	.73	−36.94 to 24.98		
Cortical CSA, mm^2^						
LMMS	242.9 (208.5 to 274.8)	247.2 (220.5 to 274.1)	.53	−12.14 to 21.94	.40	−19.43 to 25.38
Placebo	258.2 (218.5 to 285.3)	254.3 (241.6 to 273.3)	.98	−16.75 to 15.24		
Cortical thickness, mm						
LMMS	3.4 (3.3 to 3.7)	3.8 (3.4 to 3.9)	.07	−0.22 to 0.53	.18	−0.25 to 0.63
Placebo	3.8 (3.2 to 4.2)	3.8 (3.6 to 3.9)	.75	−0.32 to 0.21		
Cortical section modulus, mm^3^						
LMMS	2348.7 (2013.9 to 2683.4)	2299.3 (1955.3 to 2627.2)	.85	−159.09 to 179.48	.93	−280.84 to 138.31
Placebo	2289.7 (1977.7 to 2603.0)	2381.6 (2148.0 to 2615.4)	.71	−54.10 to 102.76		
Maximum muscle CSA, mm^2^						
LMMS	5118.6 (4882.3 to 5525.8)	5249.2 (4809.4 to 5639.1)	.67	−190.06 to 695.39	.58	−481.44 to 572.41
Placebo	5232.6 (4810.0 to 5763.3)	5415.9 (5097.1 to 5711.0)	.71	−304.10 to 498.86		
Strength-Strain Index, mm^3^						
LMMS	1909.9 (1665.5 to 2254.3)	1971.3 (1648.7 to 2252.5)	.83	−153.74 to 134.31	.83	−279.39 to 126.27
Placebo	1927.8 (1766.6 to 2208.0)	2032.8 (1826.6 to 2250.1)	.64	−68.68 to 172.33		
**DXA measurements of body composition and bone density** ^b^						
Lean body mass, kg						
LMMS	33.7 (30.5 to 37.4)	34.3 (33.0 to 35.2)	.30	−1.52 to 3.58	.63	−3.91 to 3.55
Placebo	33.8 (31.5 to 36.1)	35.5 (33.0 to 35.2)	.54	−0.76 to 3.38		
Fat mass, kg						
LMMS	10.6 (8.2 to 13.6)	12.2 (11.7 to 12.8)	.04	0.19 to 3.32	.66	−4.19 to 1.05
Placebo	10.9 (8.9 to 12.4)	12.8 (12.4 to 13.7)	.004	0.37 to 4.19		
Fat, %						
LMMS	23.8 (21.0 to 27.7)	25.2 (24.4 to 26.2)	.47	−1.49 to 3.83	.64	−3.43 to 3.40
Placebo	24.1 (20.1 to 27.2)	25.5 (25.1 to 26.0)	.12	−0.83 to 3.99		
Total body BMC, g						
LMMS	1690.7 (1539.6 to 1969.0)	1783.2 (1721.4 to 1919.6)	.42	−162.15 to 208.72	.42	−236.26 to 221.71
Placebo	1796.1 (1562.1 to 2043.3)	1877.1 (1782.1 to 1951.1)	.36	−67.28 to 107.79		
Total body *z* score						
LMMS	−0.4 (−1.2 to +0.0)	−0.4 (−0.9 to −0.3)	.50	−0.91 to 0.82	.88	−1.14 to 0.78
Placebo	−0.5 (−1.1 to +0.7)	−0.4 (−0.5 to −0.3)	.68	−0.64 to 0.45		
Hip BMC, g						
LMMS	28.0 (24.4 to 31.1)	28.3 (26.7 to 30.8)	.85	−1.42 to 4.26	.31	−2.48 to 4.13
Placebo	28.0 (24.6 to 33.8)	27.6 (26.0 to 29.4)	.91	−2.17 to 1.44		
Hip BMD, g/cm^2^						
LMMS	0.9 (0.8 to 1.0)	0.9 (0.8 to 0.9)	.98	−0.06 to 0.08	.54	−0.07 to 0.09
Placebo	0.9 (0.8 to 1.0)	0.9 (0.9 to 1.0)	.94	−0.04 to 0.04		
Hip BMD *z* score						
LMMS	−0.6 (−1.1 to 0.0)	−0.4 (−0.8 to −0.3)	.91	−0.52 to 0.36	.85	−0.81 to 0.37
Placebo	−0.4 (1.1 to +0.4)	−0.3 (−0.4 to −0.2)	.63	−0.33 to 0.47		
Spine BMC, g						
LMMS	47.0 (42.0 to 57.1)	46.2 (44.4 to 52.9)	.84	−3.85 to 8.27	.56	−5.30 to 10.77
Placebo	49.6 (38.7 to 56.3)	48.8 (45.8 to 51.2)	.88	−5.03 to 7.07		
Spine BMD, g/cm^2^						
LMMS	0.9 (0.8 to 1.0)	0.9 (−1.0 to −0.6)	.48	−0.02 to 0.08	.99	−0.08 to 0.09
Placebo	0.8 (−1.6 to 0.0)	0.9 (−0.7 to −0.6)	.55	−0.03 to 0.08		
Spine BMD *z* score						
LMMS	−0.7 (−1.1 to −0.1)	−0.6 (−1.0 to −0.6)	.44	−0.49 to 0.12	.61	−0.44 to 0.52
Placebo	−0.7 (−1.6 to 0.0)	−0.6 (−1.6 to 0.0)	.79	−0.29 to 0.41		

Abbreviations: BMC, bone mineral content; BMD, bone mineral density; CSA, cross-sectional area; DXA, dual energy x-ray absorptiometry; LMMS, low-magnitude mechanical signals; vBMD, volumetric bone mineral density.

^a^ Estimates adjusted for patient age, duration of anorexia nervosa, weight gain, and adherence (median platform-recorded minutes used per 3-month period).

^b^ For DXA measurements, 6-month adjusted medians are adjusted for patient age, duration of anorexia, and baseline 25-hydroxyvitamin D. Patient age and duration of anorexia were consistently significant in (most) models. Baseline 25-hydroxyvitamin D was significant in some but not all models.

**Table 3.** Bone Biomarkers at Baseline and After 3 and 6 Months of LMMS or Placebo in Young Women With Anorexia Nervosa and Those With Percentage of Median BMI of 85% or Less

Measure	Baseline median (IQR)	Adjusted median (IQR)^a^		Median change (IQR) from 0 to 6 mo	*P* value (within group)	95% CI for median difference (0-6 mo) within group	*P* value (difference in change between groups)	95% CI for difference in median change between groups
		3 mo	6 mo					
BSAP, ng/mL								
LMMS	28.0 (24.8 to 31.8)	43.3 (36.2 to 55.7)	36.0 (29.6 to 43.6)	4.5 (−0.9 to 11.3)	.02	1.42 to 10.11	.28	−7.98 to 3.77
Placebo	27.1 (24.3 to 31.9)	33.3 (13.9 to 45.8)	34.5 (31.1 to 41.3)	6.1 (13.9 to 45.8)	.04	2.46 to 10.04		
C-Telopeptides, pmol/L								
LMMS	0.5 (0.4 to 0.7)	0.5 (0.4 to 0.6)	0.5 (0.4 to 0.6)	<0.1 (−0.2 to 0.1)	.83	−0.15 to 0.12	.64	−0.26 to 0.04
Placebo	0.4 (0.3 to 0.6)	0.5 (0.4 to 0.51)	0.5 (0.4 to 0.6)	<0.1 (−0.3 to 0.2)	.28	−0.04 to 0.16		
Osteocalcin, μg/L								
LMMS	25.7 (22.0 to 30.0)	28.4 (24.8 to 31.2)	32.2 (30.0 to 37.1)	5.2 (2.1 to 12.3)	.01	2.31 to 11.66	.40	−5.61 to 7.48
Placebo	24.2 (21.0 to 29.2)	27.7 (25.7 to 31.2)	29.7 (22.8 to 33.3)	6.8 (−8.4 to +10.9)	.10	−3.24 to 8.11		
**Female adolescents with anorexia nervosa with percentage of median BMI of ≤85% (n = 21)**								
BSAP, ng/mL								
LMMS	26.3 (24.0 to 32.1)	33.9 (30.4 to 38.2)	30.4 (25.7 to 33.1)	2.4 (−2.1 to 6.7)	.39	−2.80 to 8.04	.04	0.17 to 12.89
Placebo	24.2 (25.5 to 31.4)	25.1 (24.2 to 28.4)	26.0 (22.4 to 27.6)	0.7 (−0.01 to 0.12)	.22	−1.11 to 5.03		
C-Telopeptides, pmol/L								
LMMS	0.47 (0.36 to 0.54)	0.41 (0.39 to 0.42)	0.53 (0.44 to 0.55)	0.07 (−0.01 to 0.12)	.39	−0.02 to 0.13	.70	−0.16 to 0.34
Placebo	0.41 (0.32 to 0.62)	0.41 (0.39 to 0.42)	0.46 (0.43 to 0.53)	0.06 (−0.28 to 0.14)	.85	−0.26 to 0.15		
Osteocalcin, ng/mL								
LMMS	22.1 (19.2 to 25.6)	24.9 (23.4 to 26.4)	29.2 (23.4 to 26.4)	6.1 (−0.3 to 10.4)	.07	−2.00 to 10.97	.06	−8.83 to 14.08
Placebo	24.4 (19.5 to 34.1)	25.4 (23.2 to 26.0)	25.8 (24.8 to 28.1)	1.1 (−7.7 to 3.9)	.67	−8.65 to 4.75		

Abbreviations: BMI, body mass index (calculated as weight in kilograms divided by height in meters squared); BSAP, bone-specific alkaline phosphatase; LMMS, low-magnitude mechanical signals.

SI conversion factors: To convert osteocalcin to micrograms per liter, multiply by 1.

^a^ Adjusted for patient age, duration of anorexia, weight gain from 0 to 6 months, and adherence (median platform-recorded minutes used per 3-month period).

**Table 4.** Serum Hormone Concentrations at Baseline and After 3 and 6 Months of LMMS or Placebo in Young Women With Anorexia Nervosa

Measure	Baseline median (IQR)	Adjusted median (IQR)^a^		Median (IQR) of change from 0 to 6 mo	*P* value (within group)	95% CI for median difference (0-6 mo within group)	*P* value (difference in change between groups)	95% CI for difference in median change between groups
		3 mo	6 mo					
IGF-1								
LMMS	154.0 (105.6 to 215.5)	163.3 (133.6 to 199.3)	171.9 (133.8 to 196.6)	4.5 (−15.9 to 11.6)	.67	−15.45 to 11.24	.39	−30.40 to 20.40
Placebo	161.9 (129.5 to 225.0)	167.3 (146.9 to 212.9)	172.3 (144.0 to 206.0)	2.8 (−27.6 to 3.5)	.76	−23.29 to 19.63		
Insulin								
LMMS	8.8 (5.3 to 32.9)	19.5 (13.6 to 23.7)	14.7 (12.1 to 17.5)	3.4 (−17.3 to 5.61)	.40	−9.10 to 5.16	.20	−14.96 to 17.83
Placebo	11.8 (5.13 to 28.2)	17.8 (14.1 to 21.6)	15.4 (14.3 to 16.8)	5.1 (−14.0 to 11.6)	.48	−12.63 to 10.05		
Cortisol								
LMMS	10.1 (8.4 to 14.4)	9.9 (7.8 to 13.3)	12.0 (9.5 to 14.0)	2.8 (−3.5 to 4.7)	.32	−1.66 to 4.62	.84	−5.03 to 4.29
Placebo	10.7 (7.6 to 14.0)	9.8 (8.3 to 11.1)	12.2 (7.6 to 14.0)	2.1 (−1.4 to 5.5)	.26	−0.69 to 5.32		
Adiponectin								
LMMS	20.9 (18.6 to 27.8)	21.2 (19.7 to 24.4)	20.0 (19.0 to 21.0)	−2.7 (−6.5 to 0.4)	.12	−6.15 to 0.31	.95	−6.09 to 4.88
Placebo	21.5 (18.1 to 27.8)	20.9 (19.7 to 22.2)	20.2 (19.4 to 20.9)	−1.7 (−8.3 to 1.6)	.11	−8.08 to 0.35		
Leptin								
LMMS	2.3 (2.0 to 3.8)	2.6 (2.4 to 2.7)	3.2 (1.3 to 4.3)	0.8 (−2.6 to 2.6)	.89	−2.63 to 2.57	.78	−2.60 to 3.28
Placebo	2.7 (2.2 to 3.0)	2.5 (2.4 to 2.7)	2.2 (1.2 to 3.9)	−0.2 (−2.0 to 0.6)	.65	−1.79 to 0.46		

Abbreviations: IGF-1, insulin-like growth factor 1; LMMS, low-magnitude mechanical signals.

^a^ Adjusted for patient age, duration of anorexia, weight gain from 0 to 6 months, and adherence (median platform-recorded minutes used per 3-month period). Patient age and duration of anorexia were consistently significant in most models. Adherence was significant only in some models.

## Results

### Participants

Among the 51 randomized participants, 1 withdrew before completing baseline measurements (Figure). The final sample of 50 participants (median [IQR] age, 16.3 [15.1-17.6] years; 3 [6.0%] Asian, 1 [2.0%] Black, 44 [88.0%] White, and 2 [4.0%] other race [no additional information provided]; 3 [6.0%] Hispanic and 47 [94.0%] non-Hispanic ethnicity) included 24 randomized to LMMS (median [IQR] age, 16.9 [14.7-18.9] years) and 26 to placebo (median [IQR] age, 16.3 [15.2-17.2] years). At baseline, the 2 groups were statistically similar in clinical characteristics (Table 1). During the trial, 1 participant was hospitalized for suicidal ideation and worsening depression, but no other adverse events occurred.

The cohort’s median (IQR) BMI was 17.8 (16.4-18.7), corresponding to a median (IQR) %mBMI of 87.2% (81.0%-91.6%). Although statistically similar (95% CI for difference in group medians, −8.36% to 4.74%; *P* = .42), median (IQR) baseline %mBMI was higher in the placebo group (87.9% [80.1%-92.0%]) vs the LMMS group (86.5% [11.9%]). Twenty-one participants (42.0%) were moderately malnourished (%mBMI ≤85.0%; 11 in the LMMS group and 10 in the placebo group) (eTable 1 in Supplement 2), and 8 (16.0%) were severely malnourished (%mBMI ≤80.0%; 5 in the LMMS group and 3 in the placebo group) (eTable 2 in Supplement 2).

The median (IQR) duration of AN was 6.0 (3.0-12.0) months (range, 0-120 months). For postmenarchal participants, the median (IQR) age at menarche was 12.0 (11.3-13.0) years and the duration of amenorrhea was 6.0 (3.0-12.0) months (range, 0-78 months) (Table 1).

Participants with vitamin D deficiency (baseline 25[OH]D<20 ng/mL [to convert to nanomoles per liter, multiply by 2.4) received a supplement of oral ergocalciferol, 50 000 IU weekly for 2 months, then 400 to 600 IU daily, to achieve normal levels. If insufficient (25[OH]D, 21-30 ng/mL), vitamin D_3_, 1000 IU/d orally, was recommended. 25(OH)D concentrations were remeasured at each visit until the documented concentration was greater than 30 ng/mL. The LMMS group had lower baseline 25(OH)D concentrations than placebo (median [IQR], 33.6 [23.1-42.3] ng/mL for the LMMS group vs 42.3 [33.3-49.6] ng/mL for the placebo group; 95% CI for difference in group medians, −18.68 to −2.70 ng/mL; *P* = .04). Fifteen participants (30.0%), including 10 in the LMMS group and 5 in the placebo group, had baseline 25(OH)D concentrations of 30 ng/mL or less.

### Longitudinal Assessments

Six participants randomized to receive LMMS and 4 receiving placebo discontinued participation (Figure). Baseline characteristics were similar for completers (78.4% of the cohort) and noncompleters (21.6%). Reasons for trial termination included loss to follow-up, inpatient psychiatric hospitalization, initiation of hormone therapy, and lack of time for study participation. At 3 months, median adherence with platform use measured 62.5% of the recommended amount; by 6 months, adherence decreased to 52.6%. Adherence did not vary between the trial arms. The participants’ clinicians determined their activity level; no participants were confined to long-term bed rest. Forty adolescents completed the trial.

### Weight and Body Composition

The placebo group demonstrated increases in both weight gain (median [IQR] gain, 1.6 [−1.2 to +6.01] kg; 95% CI, 0.08-5.97; *P* = .04) and BMI (median [IQR] gain, 0.5 [−0.3 to +2.1]; 95% CI, 0.07-1.65; *P* = .01). The LMMS group had a median (IQR) weight gain of 1.3 (−1.9 to +7.4) kg and median (IQR) BMI increase of 0.4 (−0.3 to +2.1), but both were nonsignificant (95% CI for difference in group medians, −1.13 to 4.92 kg for weight and −0.21 to 1.58 for BMI). Neither weight gain nor increase in BMI differed statistically between the 2 groups. We also observed changes in body composition. Total fat mass increased over 6 months (median [IQR] gain, 1.3 [−1.4 to +3.3] kg in the LMMS group [95% CI for difference within group, 0.19-3.32; *P* = .04] and 2.4 [0.2-4.4] kg in the placebo group [95% CI for difference within group, 0.37-4.19; *P* = .003]), but this gain in fat mass was not statistically different between groups. Lean body mass did not change in either group (Table 2). We separately analyzed the subsample of 21 participants with a %mBMI of 85% or less. Although weight gain in the LMMS group was higher than in the placebo group (median [IQR] gain, 2.8 [−1.3 to +7.0] kg for the LMMS group and 1.5 [−0.75 to +3.0] kg for the placebo group), neither finding was statistically significant. Similarly, no group differences in body composition were seen. We lacked sufficient power to compare groups in the subsample with a %mBMI of 80% or less (n = 8).

### pQCT and DXA Measurements

We examined pQCT measures at the tibia (a weight-bearing bone) and the radius (a non–weight-bearing bone) that was used as each patient’s internal control. Total bone vBMD changes were nonsignificant in both groups (95% CI for difference in median change between groups, −57.11 to 2.49): in the LMMS group, vBMD decreased from a median (IQR) of 313.4 (292.9-344.6) to 309.4 (290.4-334.0) mg/cm^3^, and in the placebo group, it increased from a median (IQR) of 308.5 (276.7-348.0) to 319.2 (309.9-338.4) mg/cm^3^. In the LMMS group, total CSA at the 4% tibia site increased from a median (IQR) of 795.8 (695.0-844.8) mm^2^ to 827.5 (803.0-839.4) mm^2^, whereas in the placebo group, it decreased from 847.3 (770.5-915.3) mm^2^ to 843.3 (828.9-857.7) mm^2^ (95% CI for difference in median change between groups, 2.94-162.53; *P* = .01). Similarly, median (IQR) trabecular CSA at the 4% site increased from 616.3 (534.8-672.3) mm^2^ to 649.2 (638.0-661.4) mm^2^ in the LMMS group but decreased in those randomized to placebo from 686.4 (589.0-740.0) mm^2^ to 647.9 (637.3-661.9) mm^2^ (95% CI for difference in median change between groups, 2.80-139.68 mm^2^; *P* = .02). No changes were estimated in measurements of cortical bone of the tibia, at the radius, or in muscle CSA (Table 2; eTable 3 in Supplement 2). The DXA measurements (areal bone mineral content or areal BMD) did not change in either group (Table 2).

### Biochemical Assessments

Over time, BSAP increased (LMMS: median [IQR] change, 4.5 [−0.9 to +11.3] ng/mL; 95% CI, 1.42-10.11 ng/mL; *P* = .02; placebo: median [IQR] change, 6.1 [−8.3 to +12.1] ng/mL; 95% CI, 2.46-10.04 ng/mL; *P* = .04), but the observed changes did not differ between groups (Table 3). These increases in BSAP were positively associated with changes in IGF-1 (β = 1.016; 95% CI, 0.07-0.25; *P* = .001) but were not associated with weight changes (β = 1.04; 95% CI, −0.09 to 2.18; *P* = .07). Osteocalcin increased from baseline to 6 months with LMMS (median [IQR], 25.7 [22.0-30.0] to 32.2 [30.0-37.1] μg/L; 95% CI for median difference, 2.31-11.66 μg/L; *P* = .01) but not with placebo use (median [IQR], 24.2 [ 21.0-29.2] to 29.7 [ 22.8-33.3] μg/L; 95% CI for median difference, −3.24 to 8.11 μg/L; *P* = .10). Changes in osteocalcin were not associated with change in weight or IGF-1. C-telopeptides did not change (Table 3).

In the subsample with a %mBMI of 85.0% or less (Table 3), the increase in BSAP in the LMMS group was higher than in the placebo group (median [IQR], 2.4 [−2.1 to +6.9] ng/mL vs 0.7 [−4.2 to +5.6] ng/mL; 95% CI, 0.17-12.89; *P* = .04). A higher but nonsignificant change in osteocalcin was estimated in those receiving LMMS (median [IQR] increase, 6.1 [−0.3 to +10.5] μg/L; 95% CI, −2.00 to 10.97 μg/L; *P* = .07) but not placebo (median [IQR] increase, 1.1 [−7.7 to +3.9] μg/L; 95% CI, −8.65 to 4.75 μg/L; *P* = .67); there was no significant difference between groups. Hormonal concentrations did not change over time within or across groups (Table 4).

## Discussion

In this double-blinded, placebo-controlled randomized clinical trial, an LMMS intervention led to significant increases in biochemical markers of bone formation and tibial total and trabecular CSA over 6 months. Although our pQCT measures allowed for a sensitive assessment of change in bone size, we did not observe bone loss or gain by DXA.

Weight-bearing activity stimulates bone remodeling and reduces the prevalence of osteoporosis-related fractures.^16,17,18,19^ However, the role of physical activity in AN-related bone loss is controversial due to the need to prioritize weight gain and minimize cardiovascular risk. When designing this trial, we chose to target the same bone-muscle unit stimulated by exercise in an alternative way via LMMS. We chose LMMS as a nonpharmacologic intervention that would be accessible to patients in their own homes, is associated with limited adverse effects, and would not negatively impact weight recovery. Our study’s strengths include the use of a placebo platform, electronic adherence monitoring, and enrollment of participants at different states of recovery from AN. Previous investigations of young people had shown promising results. Our group previously studied hospitalized patients with AN who were randomized to LMMS or a sham device; LMMS prevented a decline in bone turnover markers during bed rest.^13^ In another study, a heterogeneous group of 20 children with cerebral palsy was randomized to LMMS or placebo for 6 months.^12^ The mean change in tibial vBMD in children using active devices was +6.3%, whereas children receiving placebo had a −11.9% change. Young females with existing low BMD and a history of fracture^11^ who used the LMMS platform for 2 minutes daily or more had increased spinal trabecular vBMD (3.9% increase) and CSA of the femur (2.9% increase) relative to control participants and those with poor adherence. We observed smaller but promising positive effects of LMMS in our cohort. Several factors may explain this result. Baseline BMD varied across the cohort. Physical activity was difficult to capture given that most participants had been advised to limit exercise due to concerns for weight restoration. While meeting criteria for a restrictive eating disorder, our cohort was not severely malnourished, potentially attenuating the response to LMMS.

Adolescents with AN have low markers of bone formation and resorption, correlating with abnormal bone accrual. As such, we also investigated the potential effect of LMMS on bone turnover markers. Bone-specific alkaline phosphatase increased over time but similarly between the 2 groups; this change was correlated with weight gain. Our placebo group gained more weight during the trial than the LMMS group. This larger weight gain may have led to the BSAP increases and moderated the effect of LMMS on bone turnover. Osteocalcin increased in the LMMS group compared with the placebo group. Changes in bone turnover were more profound in participants with %mBMI of 85% or less, suggesting that more malnourished patients may be more responsive to the intervention.^4,20^ Our participants were older adolescents and many were postmenarchal, when bone turnover is less robust. Therefore, stable bone turnover would not be unexpected.

Given that BMD is positively associated with body weight, even at non–weight-bearing sites, another nonmechanical factor may modulate BMD. Circulating factors, such as IGF-1, are low in adolescents with AN, leading to deficits in bone formation as well as strength.^21,22,23^ IGF-1 stimulates osteoblast function and collagen synthesis and increases proliferation and differentiation of osteoblast precursor cells. In addition, IGF-1 regulation is nutrition dependent; circulating levels of IGF-1 decrease during short-term fasting and increase with nutritional repletion.^24,25^ Consistent with these data, we observed that changes in BSAP were positively associated with IGF-1.

Adipogenesis may also play a role. Low-magnitude mechanical signals suppress adiposity by redirecting marrow-derived stem cells.^26^ Adipocytes, osteoblasts, and myocytes are all derived from a common progenitor cell—a mesenchymal stem cell. LMMS appear to exert their anabolic effect on the skeletal system by suppressing the differentiation of stem cells into adipocytes. In postmenopausal women, marrow adiposity was reduced in response to LMMS.^27^ In 15- to 20-year-old females, LMMS-treated individuals showed an increase in spine BMD compared with controls and no change in visceral fat.^11^ The low intensity of the LMMS regimen does not interfere with weight restoration and indicates that the inhibition of adipogenesis occurs via pathways separate from exercise’s effects. We previously demonstrated changes in marrow fat in adolescents with AN compared with healthy control participants.^28,29^ If an LMMS intervention is able to moderate those changes, it may be a safe alternative to exercise for preventing bone loss while patients work toward weight restoration.

### Limitations

This study has several limitations. Our dropout rate was higher than anticipated, decreasing our statistical power to detect differences between groups, particularly in more malnourished participants. The intervention period was short, whereas bone remodeling is a long process. We enrolled participants with AN diagnosis by *DSM-5* criteria, and our cohort was female, cisgender, and predominantly White; therefore, our results may not be generalizable to the full spectrum of AN. Our cohort included adolescents who were older than those previously studied but younger than postmenopausal women who have been a focus of many interventional studies to date. This outpatient study was conducted in a less controlled setting than the prior inpatient study. Consequently, adherence with the intervention was fair. Improved adherence may have generated more robust effects. Lastly, although we chose a LMMS dose of 10 minutes daily,^11^ splitting the daily intervention into two 5-minute intervals may have led to improved efficacy.^30^

### Conclusions

In this randomized clinical trial of adolescents with AN, LMMS did not improve trabecular vBMD but led to improvement in tibial CSA and increases in bone turnover. Body composition during weight restoration may be affected by LMMS. Low-magnitude mechanical signals may represent a promising option for preservation of skeletal health in female adolescents with AN. However, future studies should use a longer duration of intervention, explore strategies to optimize adherence, and potentially focus on a more malnourished and/or diverse patient population, including male patients and those with different racial and ethnic backgrounds. As restrictive eating disorders continue to increase in prevalence, identifying an intervention that slows bone loss and prevents ultimate osteoporosis could have important public health implications.

## Notes

Supplement 1.
Trial Protocol

Supplement 2. **eTable 1.** Demographic and clinical data for n = 21 participants with %mBMI ≤85 **eTable 2.** Demographic and clinical data for n = 8 severely malnourished patients with %mBMI ≤80 **eTable 3.** Measures of radial pQCT at baseline and after 6 months of LMMS treatment or placebo in young women with anorexia nervosa (N = 50)

Supplement 3.
Data Sharing Statement
