# Supplementary material for: Low-Magnitude Mechanical Signals to Preserve Skeletal Health in Female Adolescents With Anorexia Nervosa: A Randomized Clinical Trial
Source: JAMA Netw Open. 2024 Oct 31;7(10):e2441779. doi: 10.1001/jamanetworkopen.2024.41779 (PMC11528308; doi:10.1001/jamanetworkopen.2024.41779)
Supplement: Supplement 1. — Trial Protocol [file jamanetwopen-e2441779-s001.pdf]

A. <u>Introduction</u>:

The peak bone mass achieved during adolescence is a major determinant of bone density and bone strength in adulthood[1,2]. Thus, any condition that interferes with bone accrual during this critical time period has important long-term health implications. Anorexia nervosa (AN), a disorder characterized by malnutrition, fear of weight gain, and amenorrhea, is becoming increasingly prevalent among adolescents[3,4]. Accompanying this disease are significant changes in the normal hormonal milieu, loss of lean body mass secondary to malnutrition, and frequent restrictions on the level of weight-bearing physical activity. Early bone loss is seen in over half of these patients[5-7]. Studies have shown that this decreased bone mineral density (BMD) often does not return to pre-illness levels even following weight restoration[4-6]. The skeletal health implications are substantial, and there is a pressing need to develop better methods to prevent bone loss before it occurs.

Hospitalization for patients with AN is frequently accompanied by bed rest; this relative immobilization is driven by the need to assure adequate caloric balance, as well as to address concerns that arise regarding cardiac status. However, bed rest has been shown to increase the risk of bone loss in a number of conditions[8,9] and may compound skeletal losses in AN. Our research has shown that even short-term immobilization leads to changes in bone turnover markers for these ill adolescents. Prospective, well-designed clinical studies are needed to optimize medical management and establish evidence-based protocols for the treatment of this disorder. The primary focus of the scientific portion of this K23 application is to conduct two randomized, controlled trials: a *prospective short-term* intervention to attempt to prevent an imbalance of bone turnover in hospitalized patients with AN, and to determine the *long-term* effects of a biomechanical intervention on skeletal health in ambulatory adolescents with AN. This innovative study will utilize a novel intervention of high-frequency, low magnitude mechanical signals (LMMS) delivered via a vibrating platform in an attempt to stimulate bone formation. Additionally, this project proposes to study mechanisms of bone loss through the use of bone biomarkers and novel imaging techniques including ^{18}F-fluoride positron emission tomography (^{18}F-PET) of the skeleton to localize of areas of acute bone formation, axial quantitative computed tomography (QCT), and peripheral quantitative computed tomography (pQCT) to quantify cortical BMD, trabecular BMD, and cross-sectional geometry measures that determine the load capacity and fracture risk of a bone.

Specific Aim 1: **To conduct a randomized, controlled trial to determine the effect of short-term LMMS on bone turnover in adolescents who are hospitalized for AN.**

<u>Hypothesis 1A</u>: Adolescents with AN (n=30) who receive daily LMMS during hospitalization will have increased areas of bone formation as evaluated by quantitative ^{18}F-PET of the axial skeleton after 5 days of relative immobilization as compared to admission. The placebo group (n=30) will exhibit no change in bone formation on ^{18}F-PET measurements obtained between admission (Day 1) and Day 5.

<u>Hypothesis 1B</u>: LMMS will be anabolic to bone, such that adolescents with AN who are randomized to LMMS treatment will have increased levels of bone formation markers and decreased bone resorption markers compared to a control group of AN patients receiving placebo treatment.

<u>Hypothesis 1C (exploratory)</u>: Normalization of nutrition-dependent hormonal concentrations [insulin-like growth factor-I (IGF-I), leptin, adiponectin] during hospitalization will directly correlate with osteoblast function and bone formation as measured by biomarkers of bone turnover and ^{18}F-PET.

Specific Aim 2: **To conduct a randomized, controlled trial to determine the differential long-term effects of LMMS on trabecular and cortical bone in ambulatory adolescents with AN.**

<u>Hypothesis 2A</u>: vBMD, as measured by pQCT at a weight-bearing site (tibia), will be greater in ambulatory adolescents with AN randomized to receive daily LMMS for 6 months compared to adolescents with AN randomized to placebo, and will be greater than baseline values, controlling for weight gain and exercise level.

<u>Hypothesis 2B</u>: Cumulative exposure to LMMS (number of total minutes spent on the platform during the trial) will be positively correlated with absolute and percentage change in vBMD of both the tibia and spine among all participants randomized to the active treatment arm.

Specific Aim 3: **To determine the long-term effects of LMMS on biochemical indices of bone remodeling in ambulatory adolescents with AN.**
Hypothesis 3A: LMMS will result in anabolic skeletal effects, as evidenced by increases in biochemical markers of bone formation [bone specific alkaline phosphatase (BSAP), osteocalcin (OC)] and a decrease in bone resorption [serum C-terminal telopeptide of type 1 collagen (CTx)] measured at repeated intervals over 6 months in the subjects receiving LMMS, both relative to baseline and as compared to the control group.
Hypothesis 3B (exploratory): Changes in biochemical indices of bone remodeling will correlate with changes in hormonal concentrations (IGF-I, leptin, adiponectin) observed over the 6 months of LMMS intervention.

B. Background and Significance

AN is a chronic illness with significant morbidity and mortality, including a major impact on bone mass during adolescence. The proposed study focuses on the short-term and long-term effect of a novel, non-pharmacologic intervention on bone markers and the prevention of deleterious changes in bone density and strength in adolescents with AN.

B1. Significance of the problem of bone loss and anorexia nervosa

AN, a disorder characterized by malnutrition, intense fear of weight gain, and amenorrhea, is becoming increasingly prevalent. The onset of this disorder tends to occur during adolescence, when bone mineral accretion rates should be at their highest. Two clinical features of AN, estrogen deficiency and loss of body weight, are important risk factors for osteoporosis. Multiple studies have demonstrated that early bone loss is a frequent complication of AN, occurring in over half of patients[5,6]. The mechanisms of bone loss observed in adolescent girls with AN are complex. Identified factors that may affect attainment of peak bone mass include: low estrogen and androgen levels, decreased levels of IGF-I, increased cortisol levels, poor nutrition, family history of osteoporosis, and the low body mass that is characteristic of AN. It is likely a combination of these and other factors that leads to low BMD in young women with this disease.

In patients with AN, bone remodeling differs from that observed in other estrogen-deficient states. The pathogenesis of bone loss in adolescents with AN is characterized by both impaired bone formation and accelerated bone resorption[10-12]. These abnormalities improve with refeeding; weight gain is associated with normalization of both elevated bone resorption and depressed bone formation[13]. BMD is also adversely affected. Studies have shown a significant reduction in bone mass in both trabecular and cortical bone compared with age-matched controls, with the lumbar spine (a site consisting primarily of trabecular bone) appearing to be particularly vulnerable[11,14,15]. BMD in adolescents with AN is correlated with BMI, age at onset of illness, age at menarche, lean body mass, and duration of illness[10,14,16-19]. Of significant concern is the fact that the decreased BMD that is observed often does not return to pre-illness levels even years after recovery and weight restoration[11,20-24]. Thus, the bone loss seen in this disease can represent an irreversible medical complication. The implications for life-long skeletal health are substantial; patients with AN have a seven-fold increased incidence of spontaneous fractures, which may occur at multiple sites[20]. Therefore, there is a pressing need to develop better methods for the prevention of bone loss before it occurs.

B2. Current strategies for the prevention and treatment of bone loss in adolescents with AN

The treatment of low BMD in adolescents with AN is an area of controversy and fervent research. To date, there appear to be few consistently effective strategies for either prevention or treatment of bone loss in this population. Several prospective studies have investigated the efficacy of estrogen therapy as a possible intervention. A one-year prospective study in adolescents with AN randomized patients to estrogen-progestin or placebo; no difference between groups in absolute BMD or net change in BMD at the lumbar spine or the femoral neck was noted[25]. Similar randomized, prospective investigations have found similar results[12,26,27]. These data were summarized in a recent large meta-analysis that concluded that evidence of a positive effect of estrogen HRT as a sole therapy was limited in AN[28].

Dehydroepiandrosterone (DHEA) is another hormonal modality which has been studied by our group for the prevention of bone loss. DHEA is a precursor of both estrogens and androgens, and is often decreased in patients with AN. Short-term treatment with oral DHEA has been shown to decrease markers of bone resorption and increase markers of bone turnover in adolescents with AN[29]. DHEA therapy has also been

compared to combination hormonal therapy (20 mcg ethinyl estradiol and 0.1 mg levonorgestrel)[26]. After one year of treatment, both groups exhibited significantly reduced markers of bone resorption, and the DHEA group also showed increased markers of bone formation. During treatment, maintenance of both hip and spinal BMD was seen, but there was no significant increase after accounting for weight gain. In addition, young women receiving DHEA treatment also showed improvement in psychological measures[26].

Researchers have also investigated the effect of IGF-I on bone turnover markers and BMD in young women with AN. IGF-I stimulates osteoblast function and collagen synthesis, and is abnormally low in patients with AN[10,15]. Short-term treatment for 6 days with recombinant human IGF-I (rhIGF-I) in young women with AN led to a dose-dependent increase in markers of bone turnover[30]. Subsequently, the authors conducted a randomized trial of longer duration to determine whether rhIGF-I would lead to increases in bone density in women with AN[27]. Sixty women, ages 18-38 years, with low baseline BMI and spine BMD T-scores were enrolled. Participants were randomized to one of four groups: rhIGF-I and OCP (35 mcg ethinyl estradiol, 0.4 mg norethindrone), rhIGF-I alone, OCP alone, or placebo. Spinal BMD increased in all women receiving rhIGF-I as compared to those not receiving this agent. No change in spinal BMD was seen in patients receiving OCPs vs. non-OCP users. The greatest increase in bone density was observed in the combined treatment group (rhIGF-I and OCP) compared to those receiving placebo (1.8% $\pm$ 0.8% vs. -1.0% $\pm$ 1.3%, $p<0.05$). While women treated with rhIGF-I demonstrated beneficial changes in BMD, anti-resorptive treatment with OCPs alone was not sufficient to improve BMD in these undernourished women, but appeared to augment the effects of rhIGF-I when used in combination[27]. Proof of concept regarding the beneficial skeletal effects of combined antiresorptive and anabolic therapies in AN was suggested by these data. Further research is needed regarding patient acceptance of a subcutaneous medication, and the administration of this agent to a young adolescent age group.

Preliminary research has investigated the use of bisphosphonates for treatment of bone loss and maintenance of BMD in adolescents with AN. Bisphosphonates are inhibitors of osteoclast-mediated bone resorption, and have been used to treat the low bone mass associated with cerebral palsy, osteogenesis imperfecta, and steroid use[31-33]. However, the bone loss suffered by patients with AN is related not only to accelerated bone resorption, but also to impaired bone formation. In a randomized, double-blind, placebo-controlled trial of 32 adolescents with AN, alendronate 10 mg daily was tested. After one year of treatment, no between-group difference in BMD was noted at the lumbar spine or femoral neck between treatment and placebo groups[34]. Risedronate has also been evaluated as a therapeutic agent. In a recent study, 10 women with AN and baseline osteopenia received risedronate 5 mg daily for 9 months[35]. Bone density increased significantly in patients receiving this agent as compared to control subjects, despite a lack of significant weight gain. However, the effect of bisphosphonate use in the reproductive age group is unknown, and long-term side effects of these agents have not been well-established. Further clinical studies are needed.

Although the studies of these pharmacological therapies have increased understanding of the pathophysiology of bone loss in AN, other nonpharmacologic therapies deserve critical assessment. Weight-bearing exercise that does not compromise the goal of weight gain is a promising, yet unexplored, treatment that may protect against bone loss in patients with AN. To date, the incorporation of exercise into AN treatment programs is controversial, and has not been extensively studied. LMMS may be the critical intervention which will provide a weight-bearing stimulus for skeletal formation, while minimizing risks associated with increased activity levels.

B3. Immobilization and the skeleton

Most patients with AN are placed on bed rest during hospitalization, and once discharged, exercise is prohibited or significantly restricted. However, prolonged abstinence from exercise may contribute to bone loss, increased risk of cardiac symptoms, and decreased compliance with the treatment program. Previous studies have examined the relationship between immobility and bone health in other populations. Skeletal unloading, such as occurs with bed rest, leads to reductions in the mechanical forces applied to bones. During bed rest, gravitational forces on the skeleton are 83% less than in the upright, loaded position[36]. It has been estimated that strict bed rest leads to bone loss of approximately 1-2% per month[9]. Decreased mechanical usage

depresses longitudinal growth, and stimulates bone remodeling-dependent bone loss[37]. The mechanism of this bone loss appears to be both a decrease in the bone formation rate, and a concurrent increase in the bone resorption rate[38]. Trebacz used rat models to show that during even transient immobilization, bone resorption and formation are uncoupled[39]. Osteoclastic activity increases post-immobilization, with a peak at 3 to 5 days, and is likely the major contributor to the loss of trabeculae during this rapid phase[40]. In healthy young adults, 20 days of bed rest led to both increased bone resorption and loss of BMD in lumbar and metacarpal bones[41]. Populations at highest risk for bone loss may be particularly vulnerable to changes induced by immobility. In a study comparing bone loss following ovariectomy with that following immobilization, the greatest loss of trabeculae occurred when ovariectomy was combined with immobilization[42]. This finding suggested that mechanical weight-bearing provided some protection against loss of bone through hormonal influences. Given that our adolescents with AN all have hypothalamic suppression, menstrual irregularity, and extremely low estrogen concentrations, the additive effect of bed rest may be particularly deleterious. Even short-term immobilization appears to be harmful; in healthy humans, biochemical parameters of bone turnover increase after only 7-10 days of bed rest[8,43]. Osteoclast activity appears to increase quickly; by the 2nd day of immobility, markers of bone resorption profoundly increased from baseline in healthy men placed on 6 days of head-down bed rest[9]. Our pilot data also suggest a rapid disruption of the balance of bone turnover following 5 days of bed rest for adolescents with AN (Section C1).

An additional concern is that resumption of previous mechanical loading does not seem enough to stop disuse-induced bone changes. In fact, following immobilization in rats, significant worsening of bone mechanical properties occurred during 4 weeks of full remobilization without set exercise[39]. In contrast, after 16 weeks of forelimb immobilization, young adult dogs remobilized with exercise three times/week showed complete recovery of bone mechanical properties[44]. Other animal studies corroborate the finding that exercise and remobilization are more effective than remobilization alone for restoring the normal bone trabecular network[45,46]. These collective results indicate that to prevent ongoing skeletal aberrations, the intensity of the remobilization activity must be greater than that of normal activity. Following hospitalization, exercise is prohibited for the majority of patients with AN until weight goals are reached and cardiac stability is assured; these activity restrictions are frequently long-term given the recurrent periods of relapse that our common in this population[47]. Based on these data, identifying a non-aerobic, weight-bearing intervention to protect skeletal health during this recovery time is crucial.

B4. Effect of mechanical stimulation on bone

Weight-bearing and physical activity are important mechanical stimuli for bone growth and bone remodeling, and reduce the prevalence of osteoporosis-related fractures[48]. Currently, exercise is promoted as a strategy to prevent and/or treat bone loss in postmenopausal women. The positive effects of exercise on bone are explained by Frost's 'mechanostat hypothesis', which proposes that bone strength is regulated by modeling and remodeling processes depending on forces acting on bones[49-51]. Thereby, bone is adapted to the tissue strain due to biomechanical forces. There is a positive relationship between physical activity and bone mineralization; bone mineral content (BMC) is higher with higher amounts of activity[52]. Previous studies have documented that dynamic forces, rather than static loads, are the greatest stimuli for osteogenesis[53]. In *ex vivo* work, cyclical mechanical stimulation, corresponding to physiological jumping for 5 minutes daily, resulted in increased evidence of bone formation, reduced osteocyte apoptosis, and improved osteocyte viability in human trabecular bone samples[54]. Investigations conducted in pediatric populations support these findings[55-58]. Even a brief (12 minute), high-impact weight-bearing exercise session conducted three times/week has considerable influence on BMD in early pubertal girls[57,58]. In adolescent females, resistance training led to an increase in femoral neck BMD in the intervention group[59]. Physical activity likely leads to improved bone mass in two ways. Directly, weight-loading generates compressive forces that stimulate bone accrual. Additionally, activity also indirectly promotes bone acquisition by increasing muscle mass and, therefore, increasing the forces generated on bones where muscles attach[48]. Thus, greater lean body mass would be expected to correlate with greater bone strength, consistent with our previous findings in young women with AN, and previous work that has examined the "muscle-bone unit" in adolescent and young adult populations[60,61].

The role of physical activity in AN-related bone loss is controversial. Investigators have shown no benefit of exercise[5,15,62]; harmful effects of exercise[63]; and protective effects of exercise[10,64,65]. The majority of the data

were obtained from adult studies, and are unlikely to be generalizable to young adolescents with AN. Few studies have examined the safety of prescribed exercise for AN patients. Thien et al. showed that a guided exercise program increased compliance with treatment, and did not reduce short-term gain of BMI[66]. To date, no randomized control trials of the effects of an exercise intervention on bone health in patients with AN have been carried out, due to the need to prioritize weight gain and cardiac health concerns in these patients. Our proposed LMMS intervention offers a safe, non-aerobic alternative to traditional weight-bearing exercise that will not be associated with increased metabolic demands, and will have no known cardiac risk.

B5. High frequency, low magnitude mechanical signals (LMMS)

A common perception of the skeletal response to exercise is that the mechanical load must be great to increase bone mass. However, extremely low-level (<<100 microstatin) high frequency (10-90Hz) strains on bone mass, similar to those caused by muscle contractions during postural control, have recently been demonstrated to be anabolic to bone tissue[67]. Preliminary data in animal models and human subjects has demonstrated that high frequency LMMS delivered by means of a vibrating platform can inhibit bone loss and preserve BMD in at-risk populations[68-73]. Initial studies were conducted in adult sheep that were vibrated for 20 minutes/day, 5 days/week[74]. Using QCT, the investigators observed a 34.2% increase in trabecular density of the femur in the mechanically stimulated sheep compared with control sheep. Histomorphometric analysis revealed that this effect was primarily achieved by increases in trabecular number and trabecular bone volume. The trabecular bone in the stimulated animals was 12% stiffer and 27% stronger, thus indicating that the stimulus not only improved the quantity of the bone, but also bone quality[74]. No differences were seen at the radius, indicating that the anabolic effect was specific to the skeletal region that was subjected to the mechanical signal. Short-term investigations in rats demonstrated that LMMS 10/minutes daily successfully inhibited disuse osteopenia, whereas 10 minutes/day of normal weight-bearing activity failed to curb this loss[68]. The mechanism by which LMMS causes these positive changes in the musculoskeletal system is unknown.

The efficacy of LMMS has also been demonstrated in human populations. Rubin et al. randomized 70 postmenopausal women to receive LMMS (0.2g, 30Hz) or placebo for 12 months[73]. After 12 months, the placebo group had a 3.3% loss of BMD at the lumbar spine and 2.9% loss of BMD in the trochanteric region of the femur. Over the same time period, the experimental group exhibited a loss of BMD at the spine of only 0.8% (a 2.5% benefit of treatment); BMD was gained at the trochanter (3.5% benefit of treatment). Even larger benefits were seen in the women with lowest body weight (<65kg) who were considered to be at greatest risk for skeletal deficits. However, the hope is that use of LMMS not only treats existing skeletal fragility, but also reduces the risk of osteoporosis and fractures later in life. Accordingly, studies have also been conducted in pediatric and young adult populations. A heterogeneous group of 20 pre- and post-pubertal children with cerebral palsy were randomized to LMMS intervention (0.3g, 90Hz) or placebo (10 minutes/day, 5 days/week) for 6 months[69]. Pre- and post-trial proximal tibial and spinal (L2) vBMD was measured by QCT. Over the 6-month trial, the mean change in tibial vBMD in children who stood on active devices was +6.3%, while children who stood on placebo devices had a -11.9% change in tibial vBMD. At the spine, the net benefit of treatment as compared with placebo was +6.7 mg/mL. Gilsanz et al. investigated whether brief, daily exposure to extremely low-level mechanical stimuli was anabolic to musculoskeletal development in young females with existing low BMD and a history of fracture[72]. Subjects were assigned to either a placebo or intervention group, and instructed to stand on the platform for 10 minutes/day for 12 months. Measurements of bone volume and density were obtained by QCT of the lumbar spine at baseline and 12 months. Subjects who used the platform for at least 2 minutes/day had increased spinal trabecular vBMD (3.9% increase) and cross-sectional area of the femur (2.9% increase) over control subjects and poor compliers. Additionally, intervention subjects had approximately a 5% increase in the cross-sectional area of paraspinous and thigh musculature compared to the control group. No significant adverse events were reported.

To deliver the LMMS, a small platform has been designed to deliver foot-based whole body vibration via a vertical, sinusoidal acceleration. The peak acceleration is 0.3g (1g=Earth's gravitational field); frequency is 30 Hz. Based on previous research, this dose appears to safely maximize the intervention's benefit while minimizing the amount of time required to use the device[72,75]. This acceleration is well below International Organization for Standardization and Occupational Safety and Health Administration recommendations for human limits of vibration exposure[76]. At these levels, standing humans could be safely exposed for up to 4

hours per day. Each device is equipped with a built-in electronic monitoring system that automatically records the duration during which the device is used each day and can be helpful in monitoring a subject's adherence. In studies conducted in both pre- and post-menopausal women, no adverse events were reported[69,72,73].

B6. Possible mediators of the effect of malnutrition and immobilization on bone

Although weight gain appears to reverse abnormal alterations of bone turnover in patients with AN[13], both the acute mechanisms behind these changes and the chronology of bone remodeling events remain unclear. Accompanying the malnutrition associated with AN are significant changes in the normal hormonal milieu, including estrogen deficiency, decreased androgens, increased cortisol, and growth hormone resistance. Some combination of these abnormalities is likely responsible for the early bone loss seen in these patients[5-7].

One potential mechanism to explain the effect of refeeding and immobility on bone turnover is acute changes in IGF-I concentrations. IGF-I acts upon cells of osteoblast lineage to stimulate collagen synthesis, and to increase proliferation and differentiation of osteoblast precursor cells[77]. The regulation of IGF-I is nutrition-dependent; circulating levels of IGF-I decline during acute fasting, and increase with nutritional repletion[78-82]. After only 4 days of fasting, IGF-I concentrations decrease in parallel with a reduction in indices of both bone formation and resorption[83]. Other researchers have also shown the association between diminished IGF-I concentrations, states of low bone turnover, and significant bone loss[17,80]. Chronically malnourished patients, such as women with AN, also have much lower serum IGF-I and IGFBP-3 concentrations than healthy subjects[10]; these proteins show a strong correlation with BMI[82,84]. After short-term refeeding or weight recovery, IGF-I levels increase, and have been shown to be similar to a normal comparison group[13,78,84].

IGF-I also appears to play an important role in bone turnover. To examine whether IGF-I was, in fact, an important mediator of the effect of fasting on bone turnover, subcutaneous rhIGF-I was administered to a small group of healthy females during a 10-day fast[81]. The group treated with rhIGF-I had a significant increase in markers of bone formation while continuing to fast. Control subjects showed a persistent suppression of bone formation during the entire fasting period. There was no difference in bone resorption markers between the two groups. The authors concluded that rhIGF-I administration disrupted the balance of bone turnover, and was a potent, selective osteoblast stimulator in states of acute undernutrition[81]. These findings contrasted with those from studies of postmenopausal women, in whom indices of bone formation and resorption both increased in response to 6 days of rhIGF-I administration[85]. It is possible that the estrogen deficient state of the postmenopausal subjects potentiated the effects of IGF-I to stimulate osteoclasts and enhance bone resorption[86]. Additionally, skeletal unloading appears to induce resistance to IGF-I with respect to bone formation, by inhibiting the activation of IGF-I signaling pathways, resulting in decreased proliferation of osteoblasts and their precursors[87].

Given that BMD is positively associated with body weight, even at non-weightbearing sites, it has also been suggested that a non-mechanical factor such as an adipocyte-derived hormone may modulate BMD[88]. Thus, we will explore the association between the adipocyte-derived hormones leptin and adiponectin with the skeletal assessments obtained in the proposed study. Leptin is involved in the regulation of food intake and energy metabolism. Peripherally, leptin modulates insulin sensitivity; high leptin triggers insulin resistance[89]. This hormone has garnered recent interest because of its possible role in the pathogenesis of bone loss in patients with AN[90]. Women with AN have decreased levels of leptin compared with healthy control subjects[91-93]. Nutritional rehabilitation and weight gain results in increased leptin concentrations[91,94]. Serum leptin levels were strongly correlated with bone formation markers, suggesting that a potential cause-and-effect relationship exists[94]. Leptin has also been shown to be negatively correlated with BMD at the lumbar spine and total body in perimenopausal women[95]. We hypothesize that weight-gain, both short- and long-term, will lead to increased levels of leptin and correlate with increased bone formation biomarkers. Our proposed intervention itself may also affect long-term leptin secretion. Rat studies utilizing LMMS technology demonstrate that adiposity is suppressed in mice randomized to LMMS daily vs. control mice. After 12 weeks, fat volume in the torso of LMMS mice was 27% lower than control mice, independent of differences in body mass or food intake[96]. Circulating levels of leptin were 38% lower in the LMMS group compared to controls[96]. We plan to monitor leptin concentrations every 6 months during the longitudinal phase of the study, to determine whether differences exist between the intervention and control groups.

Adiponectin is a second adipose-derived protein that is involved in energy homeostasis, and lipid and glucose metabolism[97]. Adiponectin production increases during short-term fasting[98], and rapidly decreases during refeeding[99]. Adiponectin and its receptors are known to be expressed in osteoblasts[100]. Studies show that adiponectin stimulates the proliferation, differentiation, and mineralization of osteoblasts[100-102]. In rat models, adiponectin-adenovirus treatment led to increased trabecular bone mass, decreased number of osteoclasts, and decreased bone resorption markers[103]. Adolescents with AN have higher levels of adiponectin than healthy control subjects, even after controlling for fat mass[90,91,93]. After weight restoration, adiponectin concentrations decline to levels matching those of control subjects[104]. Many investigations have demonstrated that increasing levels of adiponectin are independently associated with decreasing BMD[90,105,106].

B7. Skeletal Assessment Tools

Biochemical markers of bone turnover are molecular entities, measured in serum or urine, that offer a dynamic means for the assessment of bone resorption and bone formation. While older markers suffered from both a lack of specificity and large intra-individual variability, the newer markers offer a much more specific, direct, and precise measurement of the bone remodeling process[107]. Clinically, bone biomarkers are useful for measuring response to antiresorptive therapy, and can predict future fracture risk independent of bone density[107,108]. The proposed study will utilize several of the newest biochemical markers to quantify changes in bone turnover that occur as a result of the LMMS intervention. These indirect markers will provide a more immediate view of the effectiveness of our intervention within a short time frame[108]. We will utilize serum rather than urine samples to minimize changes in markers related to ongoing changes in both muscle mass and urinary excretion that may occur with refeeding[13]. Serum BSAP is a tetrameric glycoprotein found on the cell surfaces of osteoblasts. As an indicator of osteoblastic activity, BSAP provides information on bone formation. Serum OC is a small, noncollagenous protein that is predominantly synthesized by mature osteoblasts during bone formation, but is also released into the circulation from the matrix during resorption[109]. OC is a valid marker of bone turnover when resorption and formation are coupled, and is a specific marker of bone formation when formation and resorption are uncoupled[110]. Therefore, OC is truly a marker of bone turnover rather than a specific marker of bone formation. Lastly, we will measure serum CTx. This specific marker of bone resorption offers an advantage over older urine assays of bone resorption because of its increased sensitivity and specificity[110,111]. These measured telopeptide fragments are specific to degradation of mature bone collagen, assuring that the marker reflects the bone resorption process[107].

^{18}F-PET is a non-invasive imaging technique that allows assessment of bone metabolic activity, quantification of bone blood flow, and estimation of osteoblastic activity[112,113]. The ^{18}F-fluoride radionuclide bone tracer is extracted by bone tissue largely in proportion to bone blood flow, enters and binds to bone tissue, and labels the cells involved in bone turnover[112]. It appears specific for osteoblasts, and thus bone formation[114]. ^{18}F-PET offers the advantage of quick tracer uptake, allowing scans to be completed within 1 hour of tracer injection, and the ability to perform regional rather than global measurements[113]. We will calculate the signal intensity over a region of interest (spine and hip) using the standard uptake value (SUV), which is the most widely used parameter for quantification of PET studies in practice[115]. This measure represents the tissue activity within a region of interest corrected for the injected activity and for patient weight. Only static PET images, *without* concurrent blood sampling, are required. SUV strongly correlates with dynamic measures of bone metabolism (r=0.95), making it an easy-to-obtain surrogate endpoint for dynamic measures[115].

While the “gold standard” method for measurement of bone turnover is bone biopsy and histomorphology, quantitative ^{18}F-PET can provide comparable data without an invasive procedure. Studies comparing bone histomorphology analyses show excellent correlation with ^{18}F-PET results[116,117], indicating that ^{18}F-PET is a noninvasive measurement of bone metabolic activity. Use of quantitative ^{18}F-PET has been demonstrated in many populations[116,118]. In a study of patients with renal osteodystrophy, calculated rate of incorporation of 18F-fluoride into bone correlated with serum markers of bone turnover, and bone histomorphometry[116]. ^{18}F-PET will provide direct evidence for regional bone formation, and enable us to test our hypothesis that LMMS will lead to increases in bone formation compared to placebo treatment. Bones with the highest proportion of trabecular (cancellous) bone are at the highest risk for disuse osteoporosis. These areas are also eight times more active than cortical bone, and response to metabolic changes is faster. Just as the skeleton does not show uniform loss of bone during immobilization[40], we do not anticipate that increases in bone formation

induced by LMMS will be equally distributed throughout the skeleton. We anticipate that bone formation will be enhanced at areas of primarily trabecular bone (e.g., lumbar vertebrae and hip intertrochanteric region). We will correlate the ^{18}F-PET data with the biomarkers of bone turnover, which will provide auxiliary evidence to support our hypothesis.

Peripheral QCT (pQCT) also allows for selective measurement of trabecular and cortical vBMD, and the other cross-sectional geometry measures. Additionally, pQCT minimizes radiation exposure as measurements of the appendicular skeleton are obtained. pQCT measurements will be obtained at the proximal radius at the 20% site. This location has the most normative data in adults, and growing data are available for pediatric patients. At the tibia, we will measure the 66% site to obtain information not only about BMD at the diaphysis but also to quantify muscle mass; this location represents maximal muscular cross-section. We will also measure the 4% site at both the radius and tibia to obtain information about trabecular bone and skeletal changes that may occur at the metaphysis. Pediatric reference values for these measurements have been established by investigators using the same model scanner as in our GCRC. In addition, in a combined institutional effort, normative data for our site are being collected for use in this and other clinical studies. Imaging both the tibia (a weight-bearing site) and radius (a non-weight-bearing site) will provide us with a built-in “control” for the intervention subjects to help us determine whether skeletal changes noted over the course of the study are, in fact, related to our LMMS intervention.

Dual energy x-ray absorptiometry (DXA) has been the most widely used tool for assessment of bone mass in clinical practice. DXA measures bone in two dimensions, and allows for calculation of areal BMD. Although DXA measures are highly correlated with bone strength, strength depends on skeletal properties such as geometry, elasticity, and internal architecture, which are not reflected directly in DXA measurements. While material strength cannot yet be measured *in vivo* by non-invasive means, geometry can be measured by the means described above, and may reveal strength deficits not readily evident in conventional BMD or BMC Additionally, DXA is strongly influenced by bone and body size. However, DXA is the preferred method for measurements of body composition, and total lean body mass DXA measurements appear to be a reliable surrogate for skeletal load[119]. Thus, DXA scans of the total body will be obtained to measure ongoing changes in body composition that occur over the longitudinal study.

B7. Summary: Significance of the Proposed Research

In summary, low bone mass is a widespread, chronic source of morbidity for adolescents and young women with AN. These patients have numerous risk factors for skeletal deficits, including low body weight, poor nutritional intake, and hormonal deficits. In addition, activity restrictions are commonly imposed on these patients, both during acute hospital admissions accompanied by bed rest, and intermittently throughout their outpatient treatment course. This lack of mechanical stimulation is known to be another significant risk factor for decreased bone mass. To date, pharmacologic treatments aimed at preventing or ameliorating bone loss in this population have been met with variable results, and are not without side effects. High-frequency LMMS have been shown to have a substantial positive impact on bone quantity and bone quality in both pediatric and adult populations, including those most at risk for low BMD. Trabecular bone appears to be particularly sensitive to the positive benefits of this intervention; this area of high metabolic demand and rapid turnover is a critical “at risk” anatomic site for adolescents with AN. Additionally, muscular mass was significantly enhanced in those receiving LMMS treatment. No adverse effects of the intervention have been reported. Given the substantial benefits to overall bone health and musculoskeletal development, LMMS signals may represent the answer to the prevention and/or treatment of low bone mass in adolescents with AN: a safe, non-invasive, non-pharmacologic means to enhance both bone density and bone quality.

C. Preliminary Studies

C1. Effect of Bed Rest on Bone Turnover in Young Women Hospitalized for Anorexia Nervosa:

We recently completed an observational, prospective pilot study to determine the effect of relative immobilization (bed rest) on markers of bone turnover in adolescents and young women during acute hospitalization for medical complications of their eating disorder. We recruited participants age 13-22 years who met DSM-IV diagnostic criteria for AN, including at least 3 months of amenorrhea (n=29). All patients were placed on the CHB standardized eating disorder inpatient protocol, which includes structured refeeding guidelines and protocols for bed rest. We found that markers of bone formation (BSAP) and bone resorption (urine N-telopeptides, NTx) were both acutely suppressed (by Day 3) from baseline measures (Figure 1). However, by discharge (Day 5), an imbalance of bone turnover was seen, with an increase in NTx concentrations from the Day 3 nadir, and continued decline in BSAP levels.

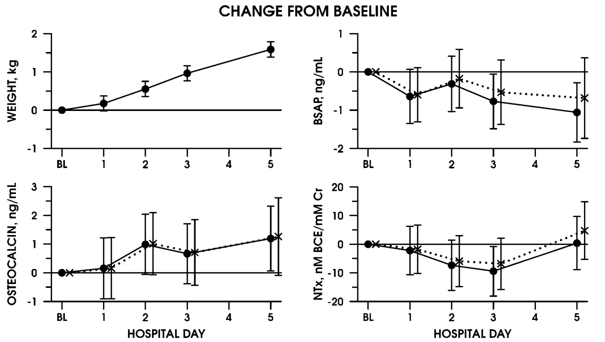

While these highly-specific markers reflect both suppressed bone formation (BSAP) and initially suppressed, then increased, resorption (NTx), a marker of the overall state of bone turnover (OC) increased from baseline to discharge, reflecting the high-turnover state provoked by bed rest and refeeding.

C2. Bone Cross-Sectional Geometry in Adolescents and Young Women with Anorexia Nervosa: A Hip Structural Analysis Study[60]:

Our group has examined hip cross sectional geometry via the Hip Structural Analysis (HSA) program in adolescents with AN. Baseline areal BMD measurements of the left total proximal femur were obtained in 85 adolescents with AN and 61 healthy control subjects by DXA. We used the HSA Program to determine BMD, cross-sectional area, and section modulus at the femoral neck and shaft. We found that femoral neck BMD was significantly lower in patients with AN compared to healthy control subjects (-36%, p<0.001); these results were replicated at the femoral shaft (-29%, p<0.001). At both regions, bone cross-sectional area and section modulus were decreased in subjects with AN compared to controls (-11% to -35%, p<0.001). In patients with AN, hip and spine BMD were correlated with cross-sectional area and section modulus at the femoral neck and shaft (r=0.27 to 0.90, p<0.01). While lean body mass was correlated with HSA variables (r=0.48 to 0.58,

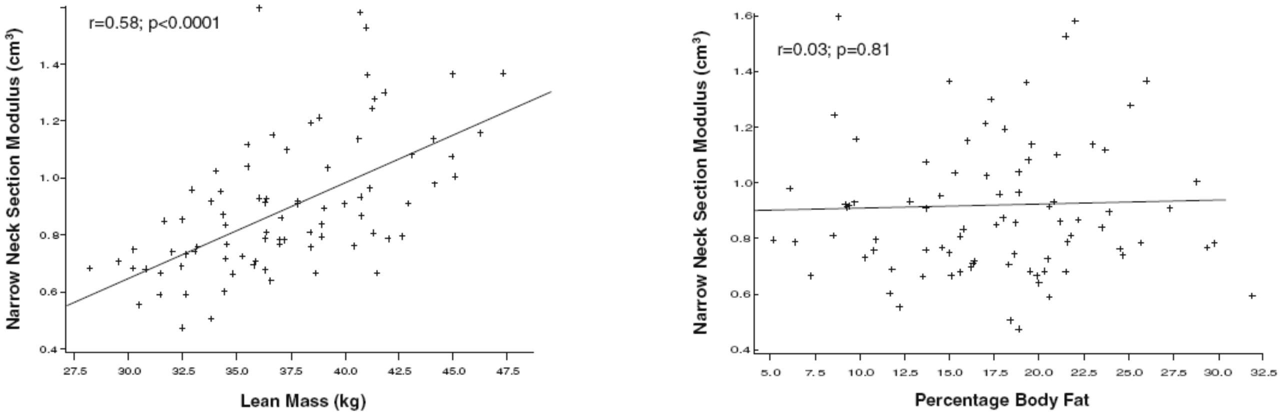

p<0.001), body fat percentage was not (Figure 2). The strong correlation between BMD and variables of hip structural geometry suggests that bone strength is compromised in these patients. Our results indicate that measures of bone geometry, as well as bone density, should be taken into account when evaluating bone health in patients with AN.

C3. Skeletal Measurements by Quantitative Ultrasound in Adolescents with Anorexia Nervosa[120]:

In this study, quantitative ultrasound (QUS) was used to evaluate skeletal status in 41 adolescents with anorexia nervosa as compared to 101

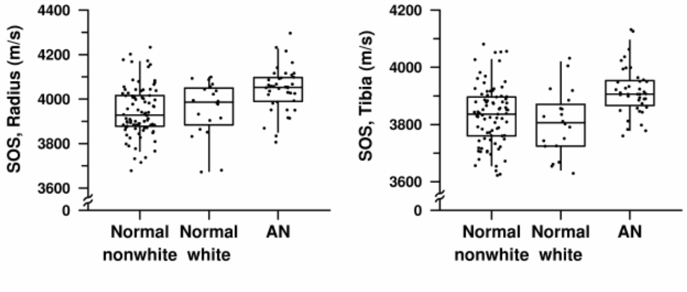

healthy control subjects. Speed of sound (SOS) was measured at the radius and tibia. Participants with AN also underwent areal BMD (aBMD) measurements by DXA of the hip and spine. Bone mineral apparent density (BMAD) was calculated to control for volumetric differences in bone size. We found that subjects with AN had higher mean SOS at the radius (4044 ± 99 m/s) than control subjects (3947 ± 116 m/s; p<0.0001) (Figure 3). These results were replicated at the tibia (AN, 3918 ± 85 m/svs. controls, 3827 ± 106 m/s; p<0.0001). Neither measures of aBMD or BMAD by DXA correlated with SOS. Weight and BMI were negative predictors of tibial, but not radial SOS. AN status remained a significant predictor of SOS after controlling for BMI, age, and race. Lastly, QUS variables did not correlate with DXA measures or anthropometric variables. We concluded that QUS is not an appropriate tool to evaluate bone density in patients with AN.

C4. Bone Density in Adolescents Treated with a GnRH Agonist for Endometriosis[121]:
In a recent study, our group examined the bone health of 36 adolescents with endometriosis (ages 13 to 21 years) who were treated with a GnRH agonist and norethindrone acetate as part of routine clinical care. In this patient cohort, the mean BMD Z-score at the total hip was -0.24 ± 1.0, with a range of -2.4 to 1.7. At this skeletal site, 17% of subjects had a slightly low BMD Z-score between -1.0 and -2.0 SD, while 6% had a significantly low BMD Z-score ≤ -2.0 SD. In comparison, the mean BMD Z-score at the lumbar spine was -0.55 ± 1.1, with a range of -2.8 to 1.4. At the spine, 31% of subjects had a BMD Z-score between -1.0 and -2.0 SD, while 9% had a Z-score ≤ -2.0 SD. Thus, while BMD at the hip was normal in the majority of the adolescents, almost 1/3 of patients demonstrated significant skeletal deficits at the lumbar spine. There was no correlation noted between duration of therapy with the GnRH agonist plus add-back and BMD at the hip or spine.

D. Research Design and Methods

D1. Study Design
We will conduct a prospective, double-blind, randomized clinical trial of a low-frequency mechanical stimulation intervention to: Aim 1) prevent an imbalance of bone turnover in adolescents hospitalized for AN; and Aim 2) determine if mechanical stimulation is an effective long-term intervention for the prevention of skeletal deficits over a 6-month period in adolescents and young women with AN.

D2. Recruitment of the Study Sample
During the first phase of the study (Years 1-3), we will recruit and enroll 60 young women hospitalized on the Adolescent Medicine service at CHB for medical complications of AN (Figure 4).

Figure 4: Phase 1, Study Overview

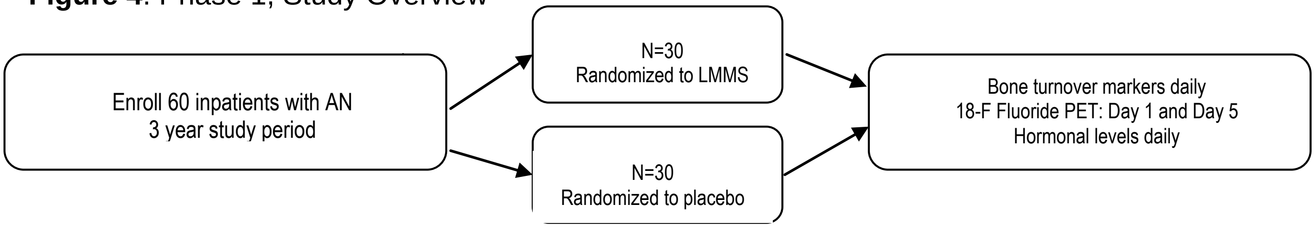

During the second study phase (Years 2-5), we will recruit 50 patients with AN to participate in an outpatient, longitudinal study of mechanical stimulation on skeletal health (Figure 5). An additional 10 patients with AN will be recruited for an outpatient sub-study targeted at obtaining compliance data during the first phase of the study (Figure 5a).

Figure 5: Phase 2, Study Overview

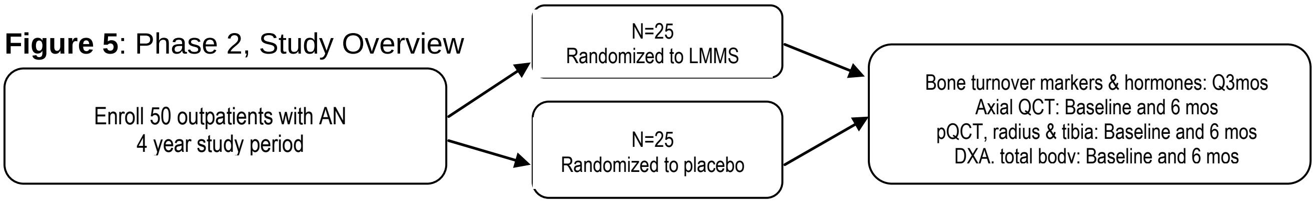

Figure 5a: Study Overview, Short-Term Compliance Study

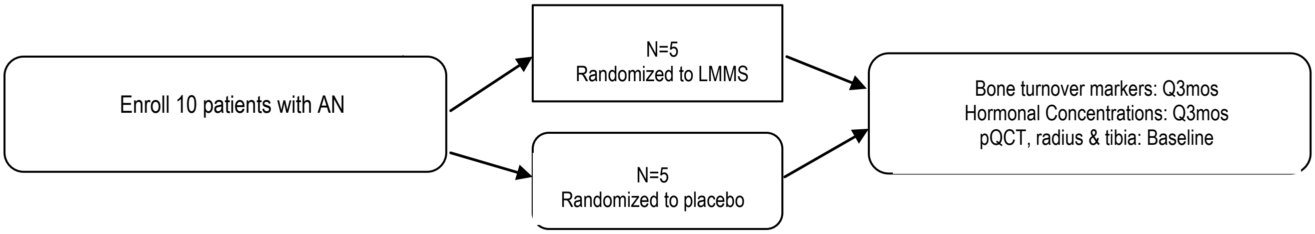

D3. Inclusion and Exclusion Criteria

D3a: Phase 1 (Inpatient Study)

Inclusion Criteria

- Age 13-22 years, at least two years post-menarche
- Patient exhibits disordered eating, distorted body image, and fear of weight gain.
- Patient has a BMI of less than 20.0 kg/m^2
- Female gender
- English-speaking
- Medical admission to the Adolescent Medicine service at Children's Hospital Boston for medical complications of anorexia nervosa, and placement on the Children's Hospital Boston Eating Disorders Clinical Practice Guideline

Exclusion Criteria

- Concomitant chronic diseases which affect bone health, such as cystic fibrosis, inflammatory bowel disease, celiac disease, renal disease, or diabetes mellitus
- Use of medications known to affect bone metabolism in the last 3 months, such as:
    - Glucocorticoid therapy (including inhaled steroids)
    - Anticonvulsants
    - Combined estrogen/progestin contraceptive agents (oral contraceptive pills)
- Depot medroxyprogesterone (Depo-Provera) use in the last 12 months
- Current pregnancy

D3b: Phase 2 (Outpatient Study)

Inclusion Criteria

- Age 11-25 years
- Patient exhibits disordered eating, distorted body image, and fear of weight gain.
- Patient has a BMI of less than 20.0 kg/m^2
- Female gender
- English-speaking

Exclusion Criteria

- Concomitant chronic diseases which affect bone health, such as cystic fibrosis, inflammatory bowel disease, celiac disease, renal disease, or diabetes mellitus
- Use of medications known to affect bone metabolism in the last 3 months, such as:
    - Glucocorticoid therapy (including inhaled steroids)
    - Anticonvulsants

- Combined estrogen/progestin contraceptive agents (oral contraceptive pills)
- Depot medroxyprogesterone (Depo-Provera) use in the last 12 months
- Current pregnancy

D4. Primary Exposure

The primary exposure will be use of a mechanical vibrating plate, the Juvent 1000 Dynamic Motion Therapy platform (FDA regulation 21 CFR 890.5380; Juvent Medical, Inc., Somerset, NJ) which provides high frequency LMMS (Image shown in Appendix D). During Phase 1, participants will be randomized to either the vibrating plate group (n=30) or to a placebo plate group (n=30). The active plate oscillates between 32-37 Hz, and is designed to create peak-to-peak accelerations of 0.3g, a dose chosen to maximize safety, efficacy, and compliance[69,72-75]. The placebo plate is identical in external appearance to the vibrating plate. It emits a high-frequency tone, identical to the noise produced by the active plate, but does not vibrate at any clinically significant frequency. Participants will stand on their assigned plate (vibrating or placebo) for 10 minutes daily for 5 days of hospitalization. Both plates will be equipped with a card reader to “log in” each participant’s daily use of the plate, and keep track of total minutes of usage per subject.

During Phase 2, participants will be randomized to either treatment with the LMMS intervention or placebo plate. Participants will be sent home with a vibrating (0.3g, 32-37 Hz) or placebo plate, and instructed to stand on the platform for 10 minutes/day for 6 months. Each plate is equipped with a monitor that tracks compliance/use of the plate, and prevents over-use of the device (i.e. the plate will not vibrate for more than the assigned time daily). At this intensity level, the motion of the active platform is barely discernible. Subject *compliance* will be assessed by an electronic monitor integrated within the device, which tabulates time, duration, and date of each treatment. After the 10 minute treatment period, the device shuts off automatically. If the subject interrupts any treatment period, the disruption is detected by the plate through a surface pressure switch. The device then emits an audible warning, and pauses until the subject returns. If the subject does not return within 10 minutes, the device will record the time activated, and shut off.

D5. Outcome Measures

The complete schedule of data collection is given in Section D7e (Phase 1) and Section D8e (Phase 2).

Specific Aim 1 is to determine the effect that LMMS during a period of relative immobilization has on bone turnover. The primary outcome measure for Phase 1 is SUV, a quantitative measurement of bone formation which will obtained by ^{18}F-PET of the axial skeleton at baseline (Day 1) and Day 5 of hospitalization. This measurement will allow us to determine whether differences in areas and amounts of bone formation exist between those inpatients randomized to treatment with LMMS vs. placebo. Secondary outcomes for Phase 1 include biomarkers of bone formation (BSAP, OC) and a marker of bone resorption (serum CTx). Hormonal concentrations will also be measured, including IGF-I and leptin. Anthropometric measurements will be obtained daily.

Specific Aim 2 is to determine the effect that longer-term LMMS have on skeletal health in adolescents with AN, over a one year period. The primary outcome for this aim is

trabecular vBMD as measured by pQCT of the tibia at baseline and 6 months. Specific Aim 3 is to determine the effect of LMMS on biomarkers of bone turnover over one year; primary outcomes are BSAP, OC, and serum CTx measured every 3 months. Secondary outcomes for Phase 2 total body DXA scans (for measurement of body composition (baseline and 6 months), anthropometric measurements, hormonal concentrations, and measures of physical activity.

D6. Covariates

Many factors may confound the relationship between AN, exercise and bone health. These include: age, BMI; dietary intake of calcium and vitamin D; duration of illness; duration of amenorrhea; previous physical activity level; smoking status; family history of osteoporosis; percentage ideal body weight; and body fat. Accordingly, these variables will be collected and accounted for in our data analyses (D7e and D8e).

D7. Logistical Issues and Data Collection: Phase 1

D7a. Patient Enrollment

A member of the study research team will be available via pager at all times, and will be contacted by the clinical care team when an adolescent with AN is admitted to the medical service at CHB. The research team member will confirm patient eligibility for possible participation in the inpatient study. Once the inclusion criteria have been confirmed, we will obtain participant consent or participant assent/parental consent (if the subject is a minor), and begin with the randomization process and study procedures. Subjects will be randomized by the study coordinator by use of randomization envelopes. Study subjects and the personnel measuring our objective study outcomes (i.e., laboratory technicians, nuclear medicine radiologist) will both be blinded as to treatment assignment. Participation in the study will not interfere with the patient's clinical care; advancement of nutrition and length of hospital stay will be determined solely by the clinical care team. This approach was utilized for our pilot study (C1), and was found to work well. The study team will keep a confidential recruitment log of the names and medical record numbers of all patients approached for study participation. This log will prevent the enrollment of the same subject on more than one occasion. Given the short-term, inpatient nature of Phase 1, we do not anticipate problems with subject adherence. Weekly reminder emails (Appendix A) will be sent to the on-call clinical team to facilitate subject identification and recruitment.

D6b. Laboratory Measurements

On the morning of Day 1 (the subjects' first morning in the hospital), we will measure markers of bone formation [serum OC and BSAP] and bone resorption [serum CTx]. Fasting samples will be obtained between 8-10AM, to account for changes that may occur with these markers secondary to feeding and diurnal variation[13,123]. Bone biomarkers will be repeated daily for 5 days. We hypothesize that the LMMS intervention will correct the imbalance between bone formation and resorption seen in our pilot study. Measurement of serum 25-hydroxyvitamin D [25(OH)D] and parathyroid hormone (PTH) will also be obtained at baseline to rule-out an underlying cause of abnormal bone metabolism that may alter bone turnover. Hormonal concentrations will be determined at intervals as outlined below; these will allow us to explore potential endocrine mediators of the effect between refeeding, LMMS, and bone turnover. Samples will be processed in the CHB CTSU, and stored at -80°C. Measurement techniques are described below.

Test Name	Analysis Method	Laboratory	Frequency	Intra-Assay Precision
BSAP	Chemiluminescent immunoassay	HCCL or MGH CLR	Daily	1.5-2.6%
CTx	ELISA	HCCL or MGH CLR	Daily	5.2-6.8%
Osteocalcin	ELISA	HCCL or MGH CLR	Daily	5.5-5.7%
PTH	Chemiluminescent immunoassay	CHB	Baseline	6.2%
25(OH)D	Chemiluminescent immunoassay	CHB	Baseline	7.3%
Insulin	Chemiluminescent immunoassay	HCCL or MGH CLR	Baseline, Day 5	2.0-4.2%
IGF-I	EIA	HCCL or MGH CLR	Daily	6.6-9.7%
Cortisol	Chemiluminescent immunoassay	HCCL or MGH CLR	Baseline, Day 5	4.4-6.7%
Adiponectin	EIA	HCCL or MGH CLR	Daily	5.0-5.4%
Leptin	RIA	HCCL or MGH CLR	Baseline, Day 5	5.2-7.5%

BSAP: bone specific alkaline phosphatase; CTx: serum C-terminal telopeptide of type 1 collagen; PTH: parathyroid hormone; 25(OH)D: 25-hydroxyvitamin D; IGF-I: insulin-like growth factor-I

D7c. Skeletal Assessments

All study subjects will undergo ^{18}F-PET scan of the axial skeleton (spine, hips, pelvis) at baseline and Day 5. A pregnancy test will be performed and results confirmed as negative on all study subjects prior to any PET procedures. Subjects will be administered ^{18}F-fluoride (2.2 MBq/kg) via IV injection. Thirty minutes post-injection, the subjects will be placed within the PET scanner (GE Advance NXi) and imaged in 3D PET mode for 5 minutes per bed position. In addition, a 3 minute emission scan will be obtained for attenuation correction. Two bed positions (15.4 cm axial field of view each) will be acquired over the lumbar spine and hip region. These data will be reconstructed into transverse slices using the iterative ordered-subset, expectation maximization algorithm. The decay-corrected administered dose and the subject's body mass will be entered such that the reconstructed data will be in units of the standard uptake value (SUV). Regions of interest (ROIs) will be manually defined over specified areas within the lumbar spine and the hip. The mean and maximum SUV value within the ROI will be recorded. The precision of PET (%CV) is 3%. The effective dose associated with a single ^{18}F-PET scan is 3.5 mSv. Our primary endpoint, SUV, will be calculated according to the formula SUV=A/(ID/m), where *A* is the mean tissue activity (µCi/g) within the volume of interest, *ID* is the injected dose (mCi), and *m* is the patient body weight (kg).

D7d. Other Measures

Height will be measured for each subject upon admission in a standardized fashion, using the same wall-mounted stadiometer. Weights will also be obtained each morning on the same digital scale, with patients clothed in a hospital gown, in a fasting state, after voiding. BMI will be calculated. Surveys will be used to quantify both nutritional intake and physical activity prior to admission. The Youth/Adolescent Questionnaire (Appendix E) is a detailed, semi-quantitative food frequency questionnaire, validated for

use in this age group, which will provide a detailed measure of calcium and vitamin D intake, as well as other nutrients[124,125]. Participants will also complete the Youth/Adolescent Activity Questionnaire (Appendix F), a validated and reproducible measure of typical time spent, over the past year, in various activities and team sports[126,127]. Study personnel will obtain a brief medical history from each participant, including information regarding medication use, past medical history, menstrual history, and smoking history, as these are all important potential covariates of bone health.

D7e.Summary: Data Collection, Phase 1

	Day 1 (baseline)	Day 2	Day 3	Day 4	Day 5
Bone markers	X	X	X	X	X
Hormone levels	X	X	X	X	X
PET scan	X				X
25(OH)D	X				
PTH	X				
Height/Weight	X	X	X	X	X
YAQ/YAAQ			X		

D8. Logistical Issues and Data Collection: Phase 2

D8a. Patient Enrollment

Enrollment for the outpatient phase of the study will also occur on an ongoing basis. Potential study subjects will be identified within the CHB Eating Disorder Program, from local area pediatric practices, and by local advertisements (Appendices B, C, and H). This recruitment approach has led to the successful enrollment of over 150 adolescents with AN for participation in our previous longitudinal studies. We will recruit an additional 10 patients into each group (intervention and control) to address potential loss to follow-up. Subjects will undergo the process of informed consent described above (D7a). After consent is obtained, subjects will complete a baseline study visit and will be randomized to treatment group by use of randomization envelopes. Study staff (study coordinator, PI) will be aware of the subjects' randomization status. However, our outcome measures are objective, and the personnel who analyze the objective measures will not be aware of treatment status. After the baseline visit, subjects will be instructed by the study coordinator as to use of the plate, and sent home with either the vibrating or placebo plate. To maximize participant adherence, a study coordinator will contact each subject monthly to address any participant concerns or questions.

D8b. Laboratory Procedures

All laboratory tests will be drawn at the same time of day at each study visit to account for differences in diurnal variation that occur with the markers of bone turnover. As is standard protocol in the CTSU, a pregnancy test will be performed at the baseline and 6-month visit prior to any skeletal assessments.

Test Name	Analysis Method	Laboratory	Frequency	Intra-Assay Precision
BSAP	Chemiluminescent immunoassay	HCCL or MGH CLR	Q 3 months	1.5-2.6%
CTx	ELISA	HCCL or MGH CLR	Q 3 months	5.2-6.8%

Osteocalcin	ELISA	HCCL or MGH CLR	Q 3 months	5.5-5.7%
PTH	ECLIA	LabCorp	Baseline	6.2%
25(OH)D	Chemiluminescent immunoassay	LabCorp	Baseline	7.3%
IGF-I	EIA	HCCL or MGH CLR	Q 3 months	6.6-9.7%
Adiponectin	EIA	HCCL or MGH CLR	Q 3 months	5.0-5.4%
Leptin	RIA	HCCL or MGH CLR	Q 3 months	5.2-7.5%
Insulin	Chemiluminescent immunoassay	HCCL or MGH CLR	Q 3 months	2.0-4.2%
Cortisol	Chemiluminescent immunoassay	HCCL or MGH CLR	Q 3 months	4.4-6.7%

BSAP: bone specific alkaline phosphatase; CTx: serum C-terminal telopeptide of type 1 collagen; PTH: parathyroid hormone; 25(OH)D: 25-hydroxyvitamin D; IGF-I: insulin-like growth factor-I

D8c. Skeletal Assessments

Peripheral quantitative computed tomography (pQCT): pQCT measurements of vBMD will be obtained in subjects at the mid-shaft of the tibia and the proximal radius using the Stratec XCT 3000 pQCT scanner (Orthometrix, White Plains, NY) at baseline and 6 months of treatment. Radiation dose per pQCT exam is 1.0 μSv effective dose equivalent. Average % CV is 1%. One technologist will perform all pQCT measurements for this protocol to avoid inter-operator variability.

Dual-energy X-ray absorptiometry (DXA): A DXA scan of the total body will be obtained at baseline and 6 months using the Hologic Discovery A scanner (Hologic Inc., Waltham, MA) to assess body composition [lean body mass (kg), total fat mass (kg), percentage body fat] at each time point. These measures will allow us to evaluate for changes in body composition occurring as a result of LMMS over the course of the investigation. Precision is monitored daily in the CHB DXA Center using standardized phantoms provided by the manufacturer (Hologic, Inc.); average % CV is 0.454 $\pm$ 0.004%. One technologist will perform all DXA measurements for this protocol to avoid inter-operator variability.

Biochemical markers of bone turnover: The same biomarkers of bone formation and resorption described in Section D7c will be utilized in Phase 2 of the study. Markers will be measured at 3 month intervals for the 6-month period of the LMMS intervention.

D8d. Other Measures

Height and weight will be measured at each study visit, in a standardized fashion (Section D7d). At every visit, all subjects will be asked to complete a 3-day dietary recall with one of the CTSU nutritionists to quantify their recent nutritional intake. Physical activity will also be quantified at each visit, by use of the Stanford Seven-Day Activity Recall (Appendix G)[128,129]. We will administer validated psychological instruments [Beck Depression Inventory[130] (Beck Depression Inventory-II for ages 13 and above, and Beck Youth Inventory-II for ages 11 to 13), Spielberger State/Trait Inventory[131] (an anxiety assessment), Eating Attitudes Test[132] (a tool for evaluation of body image and anorexic behavior)] upon admission and approaching the time of discharge to evaluate for

differences in subjective well-being, mood, and body image between time periods. As anorexic patients who are more psychologically ill tend to also be more physically ill, these psychological measures will allow us to assess the psychological phenotype of the study patients. We will include these measures as important covariates. Please see attached Appendix D for the individual instruments. Data will be downloaded from the LMMS plate regarding total minutes of use at the final study visit; these data will provide an assessment of subject compliance. Hormonal concentrations will be measured at 3-month intervals, to determine whether subjects' hormonal status is normalizing. Interval health history will be obtained from subjects at each study visit, include questions regarding return of menses. Study participants who are pre-menarchal will have a single X-ray of the left wrist obtained for bone age at the baseline visit, to allow for appropriate interpretation of the bone density results.

D8e: Summary: Data Collection, Phase 2

	**Baseline**	**3 months**	**6 months**
pQCT, radius	X		X
pQCT, tibia	X		X
DXA, total body	X		X
Bone age (if needed)	X		
Bone markers	X	X	X
Height/Weight	X	X	X
Dietary recall	X	X	X
Activity survey	X	X	X
Psychological instruments	X		X
Interval health hx	X		X
Compliance data	X	X	X
Hormonal levels	X	X	X

D8f: Phase 2 sub-study

As an antecedent to Phase 2 of the study, a short-term outpatient sub-study will take place during enrollment of Phase 1. A total of 10 subjects with AN will be enrolled following the same inclusion and exclusion criteria for Phase 2 [D3b]. Recruitment of patients will mirror the strategies approved for Phase 2 of the study [D8a], and participants will be randomized in the same manner as will be done in Phase 2 [D8a]. Participants will be sent home with either a placebo or vibrating (0.3g, 32-37Hz) platform, and instructed to stand on the platform for 10 minutes per day for the duration of the 3 month period. This initial sub-study will be done prior to Phase 2, and will be used to obtain the following; (a) a baseline pool of pQCT data for adolescent girls with AN; (b) an assessment of participant compliance with the platform device in our patient population; and, (c) preliminary data on the impact of LMMS intervention on bone formation and resorption markers.

Subjects will complete a baseline visit consistent with that of the baseline for Phase 2 of the study [D8a—D8e] the only exception being that the patient would not undergo a DXA scan. Ambulatory assessment and bone density markers will be taken. The subject with undergo a pQCT scan to measure bone geometry and strength. Subjects will additionally be asked questions regarding their health history, and be responsible for filling out questionnaires regarding their activity level. Participants will be asked to complete a second study visit upon the conclusion of the 3 month study period. This

visit will be identical in nature to the baseline, except that a pQCT scan will not be taken. Patients will be reimbursed $25 at the baseline visit. An additional $50 Gift Card will be allotted to participants upon completion of the 3 month trial period.

The compliance data obtained will be used to provide support for the results from Phase 2 of the study. pQCT data will be used to provide a control group for adolescent girls with AN with which to interpret our participant data.

D9. Data Management and Quality Control Methods

Data will be organized on standardized case report forms, developed in conjunction with the CHB Clinical Research Program. Confidential study identification numbers will be assigned to each subject. All potentially identifying information will be removed from the data collection forms. A key, linking the study identification number to the subjects' personal identifying information, will be kept in a secured location. The hard copies will be kept in secured research files in a locked file cabinet, and an office with a locked door. The data will be entered into an FDA-approved, CHB-operated database (RedCap) and kept in a password-protected computer file. We will use logic rules and range checks to ensure the accuracy and completeness of the database. A limited number of investigators will be obtaining study consent or subject evaluation. Data audits will be used. The principal investigator will review study folders at regularly scheduled intervals. Subject safety, including utilization of a Data and Safety Monitoring Board, is addressed in Section E.

D10. Statistical Analyses

D10a. Statistical Analyses: Phase 1

The **primary test of hypothesis** for Phase 1 is a comparison of the mean change in PET intensity between the two trial arms. PET scans from baseline and Day 5 will be evaluated using repeated measures analysis of variance (ANOVA), adding terms for trial arm and time x arm interaction. We will estimate the mean rate of change in each arm from parameters of the fitted model and compare arms, assessing statistical significance from the interaction term. We will also compare mean change in serum biomarkers of bone turnover (BSAP, OC, CTx) between the two trial arms. Each marker from all 5 days will be evaluated in turn using repeated measures ANOVA, adding terms for trial arm and time x arm interaction. We expect to demonstrate a decline in resorption markers and increase in formation markers in the LMMS arm compared with the non-intervention arm. Baseline clinical characteristics will be compared between the two groups using a two-sample t-test. Repeated measures ANOVA will be used to evaluate changes in anthropometric variables and body composition over time in the two groups. Hormonal concentrations, likewise measured at all 5 days, will be analyzed similarly.

D10b. Statistical Analyses: Phase 2

The **primary test of hypothesis** for Phase 2 is a comparison of the mean change in vTBMD, as measured by pQCT, between the two trial arms. At 6 months, we will calculate each subject's change from baseline and construct a 95% confidence interval about the mean change. We will perform **repeated-measures analysis of variance** (ANOVA) on the baseline and 6-month values together, taking account of intra-subject correlation over the two time points. **Secondary endpoints**, including other pQCT and DXA measures, will be analyzed similarly over the two time points. A per protocol analysis will also be conducted to determine whether any relationship exists between cumulative exposure to LMMS (number of total minutes spent on the platform during the trial) and absolute and/or percentage change in vBMD of the tibia, or if a threshold

response is reached. Additionally, to determine whether the vBMD response altered with compliance, the interaction between treatment group and cumulative exposure to LMMS will be entered into the model.

Baseline clinical characteristics will be compared between the two groups using a two-sample t-test. Repeated measures ANOVA will be used to evaluate changes in anthropometric variables and body composition over time in the two groups. **DXA and pQCT** measurements from baseline and end of study and **bone turnover markers** from all 3 study visits will be evaluated using repeated-measures ANOVA as above, adding terms for trial arm and time × arm interaction. We will estimate the mean rate of change in each arm from parameters of the fitted model and compare arms, assessing statistical significance from the interaction term. We expect to demonstrate a decline in resorption markers and increase in formation markers in the LMMS arm compared with the non-intervention arm. Hormonal concentrations, likewise measured at all 3 study visits, will be analyzed similarly. Invariant and time-varying covariates (Section D6) will be added to the repeated measures to test for mediation or effect modification of the intervention effect.

Missing data: Analysis will follow the intention-to-treat principle, with all data attributed to the subject's assigned treatment group regardless of whether treatment was delivered or completed. We will test for selective attrition by comparing baseline data between dropouts and those completing the trial. If no differential bias between the two arms of the trial is evident, the simple repeated measures analyses can be made using just the available data. If bias cannot be ruled out, we will make a conservative imputation, such as assigning the opposite group's mean change or assuming zero change. For repeated-measures analysis, we can use all available data, including baseline data on dropouts, without incurring bias if the data are "missing at random," i.e., the likelihood of lost data is related only to variables accounted for in regression.

D10c. Sample size and power analysis

All of our primary and secondary outcomes will be assessed with repeated-measures regression, comparing the time course of various bone measures, anthropometry, and biochemical markers between intervention and control subjects over k=2 or 3 evenly spaced time points. For purposes of power analysis we define realized gain as a subject's outcome trend, estimated by the appropriate linear contrast among the k evenly spaced measurements, multiplied by the duration of the study. For a measure taken only at the beginning and end of the study, realized gain is equivalent to the simple change. The standard error of realized gain, expressed as a percentage of the mean, is $\sigma_k = CV \times (12(k-1)/k(k+1))^{1/2}$, where CV indicates the coefficient of variation of the outcome (standard deviation for replicate measures of one subject on one occasion). The detectable difference in mean realized gain between two groups of n subjects is then $(t_{\alpha/2} + t_\beta)\ (2/n)^{1/2}\ \sigma_k$, where $t_{\alpha/2}$ and t_β are Student t-deviates corresponding to the two-sided Type I error rate (critical p-value, α) and the power of the comparison ($100\% \times (1-\beta)$). This quantity is tabulated below for the primary and major secondary outcomes, using measurement parameters drawn from the literature and our prior data. The table shows that our planned sample sizes provide sufficient power to detect differences in mean realized change comparable to the measurement precision (CV), a lower limit of what would be considered clinically significant. These estimates are conservative because covariate adjustment can be expected to further reduce residual variance.

Table. Power analysis for Phase 1 and Phase 2.

Phase	Duration	n/arm	Endpoint	Measurements	CV, % of mean	Detectable difference,* % of mean
1	5 d	30	PET	2	10.0	10.4
			BSAP	5	10.1	9.4
			Osteocalcin	5		21.8
2	6 mos	25	vBMD (pQCT)	2	0.4 – 1.7	0.5 – 1.9
			Body-mass index	3	5.0	5.1

* Difference between arms in mean realized gain detectable with 80% power using 2-sided Type I error rate 5% in repeated-measures analysis.

D11. Potential Limitations and Advantages

D11a. Generalizability: The population serviced by our clinic is not a representative sample of all US adolescents with eating disorders. However, in many respects, our demographics reflect national trends and observations. Our patients come from diverse socioeconomic backgrounds and attend variable educational centers (public, private, parochial schools). Subject recruitment will occur independently of these potentially confounding factors.

D11b. Biomarkers of Bone Turnover: Biomarkers of bone turnover are surrogate measures of bone formation and resorption. Despite significant improvements over last several years, these biomarkers still exhibit relatively wide variability, and are influenced by many external factors, including nutritional status and diurnal variation. Despite these shortcomings, they represent one of the few non-invasive tools we have to obtain a dynamic view of what is occurring at a cellular level within bone over the short-term.

D11c. Strengths: Despite these potential limitations, the proposed research plan offers a unique opportunity to investigate changes in bone health longitudinally, both in the short- and long-term. We also hope to discover the potential mechanisms contributing to skeletal changes over time. Using novel assessments of skeletal health, we will obtain data regarding not only bone density but also bone geometry and strength. The new insights gained from this project will provide a basis for future randomized trials in adolescents with AN and other pediatric patient populations at risk for bone loss.

D12. Project Timetable

		**Year 1**	**Year 2**	**Year 3**	**Year 4**	**Year 5**
**Research Aim 1**	**Short-term effects: LMMS**					
	Subject enrollment	X	X	X		
	Data collection and management	X	X	X		
	Data analyses		X	X		
	Manuscript preparation		X	X		
**Research Aims 2 & 3**	**Long-term effects: LMMS**					
	Subject enrollment	X	X	X	X	X
	Data collection and	X	X	X	X	

	management					
	Data analyses		X	X	X	X
	Manuscript preparation		X	X	X	X
**Prepare R01, revise**					X	X

E. Human Subjects Research

E1. Protection of Human Subjects

E1a. Human Subjects Involvement and Characteristics

Over 4 years, we will enroll adolescent females ages 11-22 years with a diagnosis of AN. Sixty subjects will be medically hospitalized inpatients (Phase 1), and 50 subjects will be ambulatory outpatients (Phase 2). All adolescents who meet these criteria will be recruited, as we believe that this age group is at significant risk to develop potentially lifelong skeletal complications of their illness. All subjects will undergo the full process of informed consent, in accordance with CHB IRB standards, prior to any research intervention or randomization procedure. Once consented, subjects will be randomized to receive either the LMMS intervention, or placebo (Phase 1)/no intervention (Phase 2). Subjects will undergo laboratory testing and imaging studies to evaluate skeletal health as previously described (Section D). All study procedures will take place at Children's Hospital Boston.

E1b. Sources of Materials

During the inpatient phase of the study (Phase 1), the enrolled subjects will have a blood specimen obtained each morning (for a total of 5 samples). A PET scan with ^{18}fluoride labeling will be conducted at baseline and upon discharge. During the outpatient study (Phase 2), participants will consent to a blood draw for research purposes only during their study visits, which will occur every 3 months for a total of 3 visits. Imaging studies (DXA, pQCT) will also be performed during the course of the study to assess bone formation and bone strength. During both study phases, participants will complete questionnaires regarding recent nutritional intake and physical activity level. These tests are all conducted for research-only purposes, and are above any laboratory testing or imaging studies which may be ordered by the participant's clinical team for purposes of clinical care. Neither the results of the laboratory measures, questionnaire data, nor imaging studies will be available within the participants' medical records. Study participants will not be identified by name or medical record number on any study document except the study log, which will link the participant's medical record number/name with assigned study ID number. This file will be kept separate from the other study documentation. All logs and data will be stored on password-protected computers in a locked office, or in a locked file cabinet. All research samples will be stored in a freezer in a locked room, labeled with the participant's research ID number, and date and time of sample.

E1c. Potential Risks

Risk to the study participant will be minimized as much as possible, from both the intervention and the data collection. Use of the LMMS platform requires standing for 10 minutes at a time, which could lead to dizziness, light-headedness, or syncope. The blood draw may produce pain from the needle, a slight bruise, and/or (rarely) syncope. Radiological procedures to measure body composition as determined by DXA may involve the minor discomfort of lying still on a firm surface for approximately 15 minutes.

This study involves minimal exposure to radiation. During Phase 1, participants will only undergo PET scan; the effective dose associated with a single ^{18}F-PET scan is 3.5 mSv. We have also attempted to minimize radiation exposure during Phase 2. The radiation exposure from the total body DXA is approximately 40μSV, less than 1% of natural background radiation received by a person in Boston over one year (or ~1/10 that of a chest x-ray), about comparable to that received during a round-trip trans-Atlantic plane flight. Bone strength will be determined by pQCT. The pQCT takes approximately 10 minutes to complete at each site. Radiation exposure from pQCT is also quite small, a dose of 0.3 uSv effective dose per scan site, and does not involve gonadal exposure. All efforts will be made to keep subjects relaxed and comfortable for the duration of the test.

E2. Adequacy of Protection Against Risks

E2a. *<u>Recruitment and Informed Consent</u>*

Members of the research team will obtain written informed consent from all potential study participants. If the potential subject is a minor (age <18 years), the parent or legal guardian will be required to sign the study consent form. Informed assent will be also obtained from the minor subject. If the potential subject is an adult (age >18 years), they alone will be required to sign the study informed consent form. Subjects will have sufficient time throughout the consent process to have all their questions answered, and any concerns addressed. If the subject and/or parent express any desire to discontinue the study at any point, the study will be terminated based on his or her wishes. The CHB Institutitional Review Board will approve the research protocol and all consent forms prior to enrolling any study subjects.

E2b. *<u>Protection against Risk</u>*

During Phase 1, all subjects will be monitored by the inpatient medical team on a daily basis due to the risks o refeeding. Clinical care will take priority over any study intervention or data collection. Subjects will have vital signs monitored frequently throughout the day, as is standard clinical care; if the subject is significantly hypotensive or orthostatic by blood pressure, use of the LMMS device or placebo plate will be delayed until later in the day when vitals have stabilized. The vibration induced by the plate is barely detectable, and the frequency and amplitude chosen to maximize safety. Additionally, each plate has an electronic compliance monitor which prevents the device from being used for more than the 10 minute/day recommended duration; this will prevent over-use of the intervention.

All blood draws will take place with the subject in the supine position, to minimize risk of syncope. A urine pregnancy test will be performed on all subjects prior to the skeletal imaging assessments (PET, pQCT, DXA) due to the radiation exposure accompanying these measurements. Results of this pregnancy test are confidential, and will be relayed to the study participant in private. Results will not be disclosed to a parent or guardian without permission of the study participant. This policy is outlined in the consent forms. If the participant experiences any psychological distress after completing a study questionnaire, study personnel will be available to discuss any concerns or questions that the subject has.

Confidentiality will be protected. Participants will not be identified by name or medical record number on any study data forms or lab specimens, only by their study ID number. Only study investigators will have access to the study logs and the data forms, which will be stored in a locked file cabinet in a locked office. Data collected from participants will be placed into this secured location within 24 hours of its acquisition. All staff involved in

research subject interaction and data collection will have up to date human research protection training, as is mandated by the CHB IRB.

Monitoring and reporting of adverse events will comply with the Children's Hospital Committee on Clinical Investigation and Clinical Research Center requirements. A Data Safety and Monitoring Board (DSMB) will be appointed for this protocol. During Phase 1, all participants will be on the inpatient medical unit of Children's Hospital, with 24 hour medical staff available should any medical issues arise. During Phase II, participants will be monitored closely during the 6-month study period with laboratory assessments and physical examinations at each visit. To assure patient safety, any participant who develops bone loss of >5% from baseline by the 6 month study visit will have bone density results relayed to their eating disorder specialist and/or primary care provider, and assistance in arranging specialty referrals (i.e. to the Bone Health Clinic) as needed. Bone loss will be monitored by the study DSMB. All study participants will be able to contact one of the study physicians at any time if questions or concerns arise.

No confidential information from this study or other data within a patient's medical record may be furnished to anyone unaffiliated with Children's Hospital without written consent, except as required by law or regulatory agencies (e.g., FDA).

E3. Potential Benefits of the Proposed Research to the Subjects and Others

We believe that the possibility for direct benefit exists, given the positive effects on bone density and bone strength seen with use of the LMMS device in other populations at high risk for bone deficits. Adolescents who participate in the study will receive free bone density measurements, as well as endocrinologic evaluation. Participants will be given information about their vitamin D status and bone density results, which they may elect to share with their primary care provider. If significant skeletal deficits or vitamin D deficiency is identified, study staff will obtain permission from the subject to share the relevant information with the subjects' clinical team. With the information gathered from the proposed study, we hope to develop evidence-based guidelines for the use of LMMS as an intervention to protect against deleterious changes in bone health. This study has the potential to benefit a large number of patients who suffer from AN.

E4. Importance of the Knowledge to Be Gained

Loss of bone mineral density is a well-established complication of AN, with potential long-term consequences including osteoporosis and increased future fracture risk. Both preventative and treatment strategies to date have been met with limited success. We are hopeful that use of the mechanical stimulation platform will prove a safe, effective means of improving the bone health of the many young women who suffer from this illness. Our results will also spearhead similar investigations in other at-risk populations, including pediatric patients with cystic fibrosis, chronic kidney disease, and inflammatory bowel disease.

E5. Data and Safety Monitoring Plan

During Phase 1, all subjects will be monitored by the inpatient medical team on a daily basis due to the risks o refeeding. Clinical care will take priority over any study intervention or data collection. Subjects will have vital signs monitored frequently throughout the day, as is standard clinical care; if the subject is significantly hypotensive or orthostatic by blood pressure, use of the LMMS device or placebo plate will be delayed until later in the day when vitals have stabilized. During Phase 2, participants will be monitored closely during the 6-month study period with laboratory assessments

and physical examinations every three months. To assure patient safety, any participant who develops bone loss of >5% from baseline by the 6 month study visit will have bone density results relayed to their eating disorder specialist and/or primary care provider, and assistance in arranging specialty referrals (i.e. to the Bone Health Clinic) as needed. Bone loss will be monitored by the study DSMB. All adverse events occurring throughout the course of the study will be reported by the principal investigator to both the CHB IRB as well as the DSMB, which will meet on a regular basis (every 6 months).

During the 6-month study, LMMS outpatient participants will complete a depression scale, anxiety scale, and eating attitudes test at the baseline and 6 month visits. These scales will be scored and screened at the time of the subjects' study visits. Any subject who reports a high level of depressive symptoms (defined as a Beck Depression Index score ≥20) or who circles a response other than "0" to question 9 (which addresses suicidal ideation; see Appendix D) will be referred to a mental health provider or their medical provider at the time of the study visit according to standard clinical practice. The EAT questionnaire will be flagged for scores of 20 or more and/or for positive responses to questions A-D, and the State Trait Anxiety Index will be flagged for scores of 65 or higher; the appropriate medical provider will be contacted in response to these events. Study personnel will not leave a participant alone if she expresses thoughts of harming herself. This will assure the participant's safety is the top priority.

All study participants will be able to contact one of the study physicians at any time if questions or concerns arise. No confidential information from this study or other data within a patient's medical record may be furnished to anyone unaffiliated with Children's Hospital without written consent, except as required by law or regulatory agencies (e.g., FDA).

Inclusion of Women and Minorities

This study will be conducted without regard to race/ethnicity, and we will make every effort to enroll adolescents of color. However, given the known epidemiology of AN, we expect that the majority of study subjects will be of Caucasian background. All study subjects will be female, as 90-95% of adolescents with anorexia nervosa are females[3].

Inclusion of Children

This study will include children, as we will be recruiting adolescents ages 11-22 years with a diagnosis of AN. We will not include younger patients due to the influence of age, pubertal stage, and growth on our primary outcome measures.

H. Literature Cited

1. Kanis JA. Assessment of fracture risk: Who should be screened? In: Favus MJ, ed. *Primer on the metabolic bone diseases and disorders of mineral metabolism. Fifth ed.* Washington, D.C.: American Society for Bone and Mineral Research; 2003:316-323.

2. Eastell R. Pathogenesis of postmenopausal osteoporosis. In: Favus MJ, ed. *Primer on the metabolic bone diseases and disorders of mineral metabolism. Fifth ed.* Washington, D.C.: American Society for Bone and Mineral Research; 2003:314-316.

3. Silber TJ. Anorexia nervosa among children and adolescents. *Adv. Pediatr.* 2005;52:49-76.

4. Golden NH. Eating disorders in adolescence: what is the role of hormone replacement therapy? *Curr. Opin. Obstet. Gynecol.* Oct 2007;19(5):434-439.

5. Bachrach LK, Guido D, Katzman D, Litt IF, Marcus R. Decreased bone density in adolescent girls with anorexia nervosa. *Pediatrics.* Sep 1990;86(3):440-447.

6. Grinspoon S, Thomas E, Pitts S, et al. Prevalence and predictive factors for regional osteopenia in women with anorexia nervosa. *Ann. Intern. Med.* Nov 21 2000;133(10):790-794.

7. Golden NH. Osteopenia and osteoporosis in anorexia nervosa. *Adolesc. Med.* Feb 2003;14(1):97-108.

8. van der Wiel HE, Lips P, Nauta J, Netelenbos JC, Hazenberg GJ. Biochemical parameters of bone turnover during ten days of bed rest and subsequent mobilization. *Bone Miner.* May 1991;13(2):123-129.

9. Heer M, Baecker N, Mika C, Boese A, Gerzer R. Immobilization induces a very rapid increase in osteoclast activity. *Acta Astronaut.* Jul 2005;57(1):31-36.

10. Gordon CM, Goodman E, Emans SJ, et al. Physiologic regulators of bone turnover in young women with anorexia nervosa. *J. Pediatr.* Jul 2002;141(1):64-70.

11. Soyka LA, Misra M, Frenchman A, et al. Abnormal bone mineral accrual in adolescent girls with anorexia nervosa. *J. Clin. Endocrinol. Metab.* Sep 2002;87(9):4177-4185.

12. Klibanski A, Biller BM, Schoenfeld DA, Herzog DB, Saxe VC. The effects of estrogen administration on trabecular bone loss in young women with anorexia nervosa. *J. Clin. Endocrinol. Metab.* Mar 1995;80(3):898-904.

13. Caillot-Augusseau A, Lafage-Proust MH, Margaillan P, et al. Weight gain reverses bone turnover and restores circadian variation of bone resorption in anorexic patients. *Clin. Endocrinol. (Oxf).* Jan 2000;52(1):113-121.

14. Misra M, Aggarwal A, Miller KK, et al. Effects of anorexia nervosa on clinical, hematologic, biochemical, and bone density parameters in community-dwelling adolescent girls. *Pediatrics.* Dec 2004;114(6):1574-1583.

15. Soyka LA, Grinspoon S, Levitsky LL, Herzog DB, Klibanski A. The effects of anorexia nervosa on bone metabolism in female adolescents. *J. Clin. Endocrinol. Metab.* Dec 1999;84(12):4489-4496.

16. Jagielska G, Wolanczyk T, Komender J, Tomaszewicz-Libudzic C, Przedlacki J, Ostrowski K. Bone mineral density in adolescent girls with anorexia nervosa--a cross-sectional study. *Eur. Child Adolesc. Psychiatry.* Apr 2002;11(2):57-62.

17. Biller BM, Saxe V, Herzog DB, Rosenthal DI, Holzman S, Klibanski A. Mechanisms of osteoporosis in adult and adolescent women with anorexia nervosa. *J. Clin. Endocrinol. Metab.* Mar 1989;68(3):548-554.

18. Audi L, Vargas DM, Gussinye M, Yeste D, Marti G, Carrascosa A. Clinical and biochemical determinants of bone metabolism and bone mass in adolescent female patients with anorexia nervosa. *Pediatr. Res.* Apr 2002;51(4):497-504.

19. Turner JM, Bulsara MK, McDermott BM, Byrne GC, Prince RL, Forbes DA. Predictors of low bone density in young adolescent females with anorexia nervosa and other dieting disorders. *Int. J. Eat. Disord.* Nov 2001;30(3):245-251.

20. Rigotti NA, Neer RM, Skates SJ, Herzog DB, Nussbaum SR. The clinical course of osteoporosis in anorexia nervosa. A longitudinal study of cortical bone mass. *JAMA.* Mar 6 1991;265(9):1133-1138.

21. Bachrach LK, Katzman DK, Litt IF, Guido D, Marcus R. Recovery from osteopenia in adolescent girls with anorexia nervosa. *J. Clin. Endocrinol. Metab.* Mar 1991;72(3):602-606.

22. Valla A, Groenning IL, Syversen U, Hoeiseth A. Anorexia nervosa: slow regain of bone mass. *Osteoporos. Int.* 2000;11(2):141-145.

23. Hartman D, Crisp A, Rooney B, Rackow C, Atkinson R, Patel S. Bone density of women who have recovered from anorexia nervosa. *Int. J. Eat. Disord.* Jul 2000;28(1):107-112.

24. Bass SL, Saxon L, Corral AM, et al. Near normalisation of lumbar spine bone density in young women with osteopenia recovered from adolescent onset anorexia nervosa: a longitudinal study. *J. Pediatr. Endocrinol. Metab.* Sep 2005;18(9):897-907.

25. Golden NH, Lanzkowsky L, Schebendach J, Palestro CJ, Jacobson MS, Shenker IR. The effect of estrogen-progestin treatment on bone mineral density in anorexia nervosa. *J. Pediatr. Adolesc. Gynecol.* Jun 2002;15(3):135-143.

26. Gordon CM, Grace E, Emans SJ, et al. Effects of oral dehydroepiandrosterone on bone density in young women with anorexia nervosa: a randomized trial. *J. Clin. Endocrinol. Metab.* Nov 2002;87(11):4935-4941.

27. Grinspoon S, Thomas L, Miller K, Herzog D, Klibanski A. Effects of recombinant human IGF-I and oral contraceptive administration on bone density in anorexia nervosa. *J. Clin. Endocrinol. Metab.* Jun 2002;87(6):2883-2891.

28. Liu SL, Lebrun CM. Effect of oral contraceptives and hormone replacement therapy on bone mineral density in premenopausal and perimenopausal women: a systematic review. *Br. J. Sports Med.* Jan 2006;40(1):11-24.

29. Gordon CM, Grace E, Emans SJ, Goodman E, Crawford MH, Leboff MS. Changes in bone turnover markers and menstrual function after short-term oral DHEA in young women with anorexia nervosa. *J. Bone Miner. Res.* Jan 1999;14(1):136-145.

30. Grinspoon S, Baum H, Lee K, Anderson E, Herzog D, Klibanski A. Effects of short-term recombinant human insulin-like growth factor I administration on bone turnover in osteopenic women with anorexia nervosa. *J. Clin. Endocrinol. Metab.* Nov 1996;81(11):3864-3870.

31. Munns CF, Rauch F, Travers R, Glorieux FH. Effects of intravenous pamidronate treatment in infants with osteogenesis imperfecta: clinical and histomorphometric outcome. *J. Bone Miner. Res.* Jul 2005;20(7):1235-1243.

32. Henderson RC, Lark RK, Kecskemethy HH, Miller F, Harcke HT, Bachrach SJ. Bisphosphonates to treat osteopenia in children with quadriplegic cerebral palsy: a randomized, placebo-controlled clinical trial. *J. Pediatr.* Nov 2002;141(5):644-651.

33. Allgrove J. Use of bisphosphonates in children and adolescents. *J. Pediatr. Endocrinol. Metab.* 2002;15 Suppl 3:921-928.

34. Golden NH, Iglesias EA, Jacobson MS, et al. Alendronate for the treatment of osteopenia in anorexia nervosa: a randomized, double-blind, placebo-controlled trial. *J. Clin. Endocrinol. Metab.* Jun 2005;90(6):3179-3185.

35. Miller KK, Grieco KA, Mulder J, et al. Effects of risedronate on bone density in anorexia nervosa. *J. Clin. Endocrinol. Metab.* Aug 2004;89(8):3903-3906.

36. Schneider V, Oganov V, LeBlanc A, et al. Bone and body mass changes during space flight. *Acta Astronaut.* Oct-Dec 1995;36(8-12):463-466.

37. Frost HM, Jee WS. On the rat model of human osteopenias and osteoporoses. *Bone Miner.* Sep 1992;18(3):227-236.

38. Bikle DD, Halloran BP. The response of bone to unloading. *J. Bone Miner. Metab.* 1999;17(4):233-244.

39. Trebacz H. Disuse-induced deterioration of bone strength is not stopped after free remobilization in young adult rats. *J. Biomech.* Dec 2001;34(12):1631-1636.

40. Doty SB, DiCarlo EF. Pathophysiology of immobilization osteoporosis. *Curr Opin Orthop.* Oct 1995;6(5):45-49.

41. Fukuoka H, Nishimura Y, Haruna M, et al. Effect of bed rest immobilization on metabolic turnover of bone and bone mineral density. *J Gravit Physiol.* Jan 1997;4(1):S75-81.

42. Bagi CM, Miller SC. Comparison of osteopenic changes in cancellous bone induced by ovariectomy and/or immobilization in adult rats. *Anat. Rec.* Jul 1994;239(3):243-254.

43. Lueken SA, Arnaud SB, Taylor AK, Baylink DJ. Changes in markers of bone formation and resorption in a bed rest model of weightlessness. *J. Bone Miner. Res.* Dec 1993;8(12):1433-1438.

44. Kaneps AJ, Stover SM, Lane NE. Changes in canine cortical and cancellous bone mechanical properties following immobilization and remobilization with exercise. *Bone.* Nov 1997;21(5):419-423.

45. Kannus P, Sievanen H, Jarvinen TL, et al. Effects of free mobilization and low- to high-intensity treadmill running on the immobilization-induced bone loss in rats. *J. Bone Miner. Res.* Oct 1994;9(10):1613-1619.

46. Bourrin S, Palle S, Genty C, Alexandre C. Physical exercise during remobilization restores a normal bone trabecular network after tail suspension-induced osteopenia in young rats. *J. Bone Miner. Res.* May 1995;10(5):820-828.

47. Herzog DB, Dorer DJ, Keel PK, et al. Recovery and relapse in anorexia and bulimia nervosa: a 7.5-year follow-up study. *J. Am. Acad. Child Adolesc. Psychiatry.* Jul 1999;38(7):829-837.

48. Vicente-Rodriguez G. How does exercise affect bone development during growth? *Sports Med.* 2006;36(7):561-569.

49. Frost HM. Perspectives: a proposed general model of the "mechanostat" (suggestions from a new skeletal-biologic paradigm). *Anat. Rec.* Feb 1996;244(2):139-147.

50. Schoenau E, Neu MC, Manz F. Muscle mass during childhood--relationship to skeletal development. *J Musculoskelet Neuronal Interact.* Mar 2004;4(1):105-108.

51. Specker BL, Schoenau E. Quantitative bone analysis in children: current methods and recommendations. *J. Pediatr.* Jun 2005;146(6):726-731.

52. Bailey DA, McKay HA, Mirwald RL, Crocker PR, Faulkner RA. A six-year longitudinal study of the relationship of physical activity to bone mineral accrual in growing children: the university of Saskatchewan bone mineral accrual study. *J. Bone Miner. Res.* Oct 1999;14(10):1672-1679.

53. Lanyon LE, Rubin CT. Static vs dynamic loads as an influence on bone remodelling. *J. Biomech.* 1984;17(12):897-905.

54. Mann V, Huber C, Kogianni G, Jones D, Noble B. The influence of mechanical stimulation on osteocyte apoptosis and bone viability in human trabecular bone. *J Musculoskelet Neuronal Interact.* Oct-Dec 2006;6(4):408-417.

55. McKay HA, MacLean L, Petit M, et al. "Bounce at the Bell": a novel program of short bouts of exercise improves proximal femur bone mass in early pubertal children. *Br. J. Sports Med.* Aug 2005;39(8):521-526.

56. Petit MA, McKay HA, MacKelvie KJ, Heinonen A, Khan KM, Beck TJ. A randomized school-based jumping intervention confers site and maturity-specific benefits on bone structural properties in girls: a hip structural analysis study. *J. Bone Miner. Res.* Mar 2002;17(3):363-372.

57. Mackelvie KJ, McKay HA, Khan KM, Crocker PR. A school-based exercise intervention augments bone mineral accrual in early pubertal girls. *J. Pediatr.* Oct 2001;139(4):501-508.

58. MacKelvie KJ, Khan KM, Petit MA, Janssen PA, McKay HA. A school-based exercise intervention elicits substantial bone health benefits: a 2-year randomized controlled trial in girls. *Pediatrics.* Dec 2003;112(6 Pt 1):e447.

59. Nichols DL, Sanborn CF, Love AM. Resistance training and bone mineral density in adolescent females. *J. Pediatr.* Oct 2001;139(4):494-500.

60. Divasta AD, Beck TJ, Petit MA, Feldman HA, Leboff MS, Gordon CM. Bone cross-sectional geometry in adolescents and young women with anorexia nervosa: a hip structural analysis study. *Osteoporos. Int.* Jan 5 2007.

61. Rauch F, Bailey DA, Baxter-Jones A, Mirwald R, Faulkner R. The 'muscle-bone unit' during the pubertal growth spurt. *Bone.* May 2004;34(5):771-775.

62. Hay PJ, Hall A, Delahunt JW, Harper G, Mitchell AW, Salmond C. Investigation of osteopaenia in anorexia nervosa. *Aust. N. Z. J. Psychiatry.* Jun 1989;23(2):261-268.

63. Joyce JM, Warren DL, Humphries LL, Smith AJ, Coon JS. Osteoporosis in women with eating disorders: comparison of physical parameters, exercise, and menstrual status with SPA and DPA evaluation. *J. Nucl. Med.* Mar 1990;31(3):325-331.

64. Seeman E, Szmukler GI, Formica C, Tsalamandris C, Mestrovic R. Osteoporosis in anorexia nervosa: the influence of peak bone density, bone loss, oral contraceptive use, and exercise. *J. Bone Miner. Res.* Dec 1992;7(12):1467-1474.

65. Rigotti NA, Nussbaum SR, Herzog DB, Neer RM. Osteoporosis in women with anorexia nervosa. *N. Engl. J. Med.* Dec 20 1984;311(25):1601-1606.

66. Thien V, Thomas A, Markin D, Birmingham CL. Pilot study of a graded exercise program for the treatment of anorexia nervosa. *Int. J. Eat. Disord.* Jul 2000;28(1):101-106.

67. Eisman JA. Good, good, good... good vibrations: the best option for better bones? *Lancet.* Dec 8 2001;358(9297):1924-1925.

68. Rubin C, Xu G, Judex S. The anabolic activity of bone tissue, suppressed by disuse, is normalized by brief exposure to extremely low-magnitude mechanical stimuli. *FASEB J.* Oct 2001;15(12):2225-2229.

69. Ward K, Alsop C, Caulton J, Rubin C, Adams J, Mughal Z. Low magnitude mechanical loading is osteogenic in children with disabling conditions. *J. Bone Miner. Res.* Mar 2004;19(3):360-369.

70. Garman R, Gaudette G, Donahue LR, Rubin C, Judex S. Low-level accelerations applied in the absence of weight bearing can enhance trabecular bone formation. *J. Orthop. Res.* Jun 2007;25(6):732-740.

71. Xie L, Jacobson JM, Choi ES, et al. Low-level mechanical vibrations can influence bone resorption and bone formation in the growing skeleton. *Bone.* Nov 2006;39(5):1059-1066.

72. Gilsanz V, Wren TA, Sanchez M, Dorey F, Judex S, Rubin C. Low-level, high-frequency mechanical signals enhance musculoskeletal development of young women with low BMD. *J. Bone Miner. Res.* Sep 2006;21(9):1464-1474.

73. Rubin C, Recker R, Cullen D, Ryaby J, McCabe J, McLeod K. Prevention of postmenopausal bone loss by a low-magnitude, high-frequency mechanical stimuli: a clinical trial assessing compliance, efficacy, and safety. *J. Bone Miner. Res.* Mar 2004;19(3):343-351.

74. Rubin C, Turner AS, Bain S, Mallinckrodt C, McLeod K. Anabolism. Low mechanical signals strengthen long bones. *Nature.* Aug 9 2001;412(6847):603-604.

75. Hannan MT, Cheng DM, Green E, Swift C, Rubin CT, Kiel DP. Establishing the compliance in elderly women for use of a low level mechanical stress device in a clinical osteoporosis study. *Osteoporos. Int.* Nov 2004;15(11):918-926.

76. Rubin C, Judex S, Qin YX. Low-level mechanical signals and their potential as a non-pharmacological intervention for osteoporosis. *Age Ageing.* Sep 2006;35 Suppl 2:ii32-ii36.

77. Hock JM, Centrella M, Canalis E. Insulin-like growth factor I has independent effects on bone matrix formation and cell replication. *Endocrinology.* Jan 1988;122(1):254-260.

78. Clemmons DR, Underwood LE, Dickerson RN, et al. Use of plasma somatomedin-C/insulin-like growth factor I measurements to monitor the response to nutritional repletion in malnourished patients. *Am. J. Clin. Nutr.* Feb 1985;41(2):191-198.

79. Merimee TJ, Zapf J, Froesch ER. Insulin-like growth factors in the fed and fasted states. *J. Clin. Endocrinol. Metab.* Nov 1982;55(5):999-1002.

80. Hochberg Z, Hertz P, Colin V, et al. The distal axis of growth hormone (GH) in nutritional disorders: GH-binding protein, insulin-like growth factor-I (IGF-I), and IGF-I receptors in obesity and anorexia nervosa. *Metabolism.* Jan 1992;41(1):106-112.

81. Grinspoon SK, Baum HB, Peterson S, Klibanski A. Effects of rhIGF-I administration on bone turnover during short-term fasting. *J. Clin. Invest.* Aug 1995;96(2):900-906.

82. Isley WL, Underwood LE, Clemmons DR. Changes in plasma somatomedin-C in response to ingestion of diets with variable protein and energy content. *JPEN. J. Parenter. Enteral Nutr.* Jul-Aug 1984;8(4):407-411.

83. Grinspoon SK, Baum HB, Kim V, Coggins C, Klibanski A. Decreased bone formation and increased mineral dissolution during acute fasting in young women. *J. Clin. Endocrinol. Metab.* Dec 1995;80(12):3628-3633.

84. Counts DR, Gwirtsman H, Carlsson LM, Lesem M, Cutler GB, Jr. The effect of anorexia nervosa and refeeding on growth hormone-binding protein, the insulin-like growth factors (IGFs), and the IGF-binding proteins. *J. Clin. Endocrinol. Metab.* Sep 1992;75(3):762-767.

85. Ebeling PR, Jones JD, O'Fallon WM, Janes CH, Riggs BL. Short-term effects of recombinant human insulin-like growth factor I on bone turnover in normal women. *J. Clin. Endocrinol. Metab.* Nov 1993;77(5):1384-1387.

86. Turner RT, Riggs BL, Spelsberg TC. Skeletal effects of estrogen. *Endocr. Rev.* Jun 1994;15(3):275-300.

87. Sakata T, Wang Y, Halloran BP, Elalieh HZ, Cao J, Bikle DD. Skeletal unloading induces resistance to insulin-like growth factor-I (IGF-I) by inhibiting activation of the IGF-I signaling pathways. *J. Bone Miner. Res.* Mar 2004;19(3):436-446.

88. Richards JB, Valdes AM, Burling K, Perks UC, Spector TD. Serum adiponectin and bone mineral density in women. *J. Clin. Endocrinol. Metab.* Apr 2007;92(4):1517-1523.

89. Mallamaci F, Tripepi G, Zoccali C. Leptin in end stage renal disease (ESRD): a link between fat mass, bone and the cardiovascular system. *J Nephrol.* Jul-Aug 2005;18(4):464-468.

90. Misra M, Miller KK, Cord J, et al. Relationships between serum adipokines, insulin levels, and bone density in girls with anorexia nervosa. *J. Clin. Endocrinol. Metab.* Jun 2007;92(6):2046-2052.
91. Bosy-Westphal A, Brabant G, Haas V, et al. Determinants of plasma adiponectin levels in patients with anorexia nervosa examined before and after weight gain. *Eur. J. Nutr.* Sep 2005;44(6):355-359.
92. Hebebrand J, Blum WF, Barth N, et al. Leptin levels in patients with anorexia nervosa are reduced in the acute stage and elevated upon short-term weight restoration. *Mol. Psychiatry.* Jul 1997;2(4):330-334.
93. Housova J, Anderlova K, Krizova J, et al. Serum adiponectin and resistin concentrations in patients with restrictive and binge/purge form of anorexia nervosa and bulimia nervosa. *J. Clin. Endocrinol. Metab.* Mar 2005;90(3):1366-1370.
94. Heer M, Mika C, Grzella I, Drummer C, Herpertz-Dahlmann B. Changes in bone turnover in patients with anorexia nervosa during eleven weeks of inpatient dietary treatment. *Clin. Chem.* May 2002;48(5):754-760.
95. Kontogianni MD, Dafni UG, Routsias JG, Skopouli FN. Blood leptin and adiponectin as possible mediators of the relation between fat mass and BMD in perimenopausal women. *J. Bone Miner. Res.* Apr 2004;19(4):546-551.
96. Rubin CT, Capilla E, Luu YK, et al. Adipogenesis is inhibited by brief, daily exposure to high-frequency, extremely low-magnitude mechanical signals. *Proc. Natl. Acad. Sci. U. S. A.* Nov 6 2007;104(45):17879-17884.
97. Nedvidkova J, Smitka K, Kopsky V, Hainer V. Adiponectin, an adipocyte-derived protein. *Physiol. Res.* 2005;54(2):133-140.
98. Halberg N, Henriksen M, Soderhamn N, et al. Effect of intermittent fasting and refeeding on insulin action in healthy men. *J. Appl. Physiol.* Dec 2005;99(6):2128-2136.
99. Liu YM, Lacorte JM, Viguerie N, et al. Adiponectin gene expression in subcutaneous adipose tissue of obese women in response to short-term very low calorie diet and refeeding. *J. Clin. Endocrinol. Metab.* Dec 2003;88(12):5881-5886.
100. Luo XH, Guo LJ, Yuan LQ, et al. Adiponectin stimulates human osteoblasts proliferation and differentiation via the MAPK signaling pathway. *Exp. Cell Res.* Sep 10 2005;309(1):99-109.
101. Shinoda Y, Yamaguchi M, Ogata N, et al. Regulation of bone formation by adiponectin through autocrine/paracrine and endocrine pathways. *J. Cell. Biochem.* Sep 1 2006;99(1):196-208.
102. Kanazawa I, Yamaguchi T, Yano S, Yamauchi M, Yamamoto M, Sugimoto T. Adiponectin and AMP kinase activator stimulate proliferation, differentiation, and mineralization of osteoblastic MC3T3-E1 cells. *BMC Cell Biol.* 2007;8:51.
103. Oshima K, Nampei A, Matsuda M, et al. Adiponectin increases bone mass by suppressing osteoclast and activating osteoblast. *Biochem. Biophys. Res. Commun.* Jun 3 2005;331(2):520-526.
104. Modan-Moses D, Stein D, Pariente C, et al. Modulation of adiponectin and leptin during refeeding of female anorexia nervosa patients. *J. Clin. Endocrinol. Metab.* May 2007;92(5):1843-1847.
105. Jurimae J, Rembel K, Jurimae T, Rehand M. Adiponectin is associated with bone mineral density in perimenopausal women. *Horm. Metab. Res.* May 2005;37(5):297-302.
106. Lenchik L, Register TC, Hsu FC, et al. Adiponectin as a novel determinant of bone mineral density and visceral fat. *Bone.* Oct 2003;33(4):646-651.

107. Christgau S, Cloos PA. Current and future applications of bone turnover markers. *Clin. Lab.* 2003;49(9-10):439-446.
108. Srivastava AK, Vliet EL, Lewiecki EM, et al. Clinical use of serum and urine bone markers in the management of osteoporosis. *Curr. Med. Res. Opin.* Jul 2005;21(7):1015-1026.
109. Camacho P, Kleerekoper M. Biochemical Markers of Bone Turnover. In: Favus MJ, ed. *Primer on the Metabolic Bone Diseases and Disorders of Mineral Metabolism*. Sixth ed. Washington, D.C.: American Society for Bone and Mineral Research; 2006:127-132.
110. Garnero P, Delmas PD. Biochemical markers of bone turnover. Applications for osteoporosis. *Endocrinol. Metab. Clin. North Am.* Jun 1998;27(2):303-323.
111. Woitge HW, Pecherstorfer M, Li Y, et al. Novel serum markers of bone resorption: clinical assessment and comparison with established urinary indices. *J. Bone Miner. Res.* May 1999;14(5):792-801.
112. Grant FD, Fahey FH, Packard AB, Davis RT, Alavi A, Treves ST. Skeletal PET with 18F-fluoride: applying new technology to an old tracer. *J. Nucl. Med.* Jan 2008;49(1):68-78.
113. Cook GJ, Fogelman I. The role of positron emission tomography in skeletal disease. *Semin. Nucl. Med.* Jan 2001;31(1):50-61.
114. Blake GM, Park-Holohan SJ, Cook GJ, Fogelman I. Quantitative studies of bone with the use of 18F-fluoride and 99mTc-methylene diphosphonate. *Semin. Nucl. Med.* Jan 2001;31(1):28-49.
115. Brenner W, Vernon C, Muzi M, et al. Comparison of different quantitative approaches to 18F-fluoride PET scans. *J. Nucl. Med.* Sep 2004;45(9):1493-1500.
116. Messa C, Goodman WG, Hoh CK, et al. Bone metabolic activity measured with positron emission tomography and [18F]fluoride ion in renal osteodystrophy: correlation with bone histomorphometry. *J. Clin. Endocrinol. Metab.* Oct 1993;77(4):949-955.
117. Piert M, Zittel TT, Becker GA, et al. Assessment of porcine bone metabolism by dynamic [18F]Fluoride Ion PET: Correlation with bone histomorphometry. *J. Nucl. Med.* Jul 2001;42(7):1091-1100.
118. Blake GM, Park-Holohan SJ, Fogelman I. Quantitative studies of bone in postmenopausal women using (18)F-fluoride and (99m)Tc-methylene diphosphonate. *J. Nucl. Med.* Mar 2002;43(3):338-345.
119. Petit MA, Beck TJ, Shults J, Zemel BS, Foster BJ, Leonard MB. Proximal femur bone geometry is appropriately adapted to lean mass in overweight children and adolescents. *Bone.* Mar 2005;36(3):568-576.
120. Divasta AD, Ringelheim J, Bristol SK, Feldman HA, Gordon CM. Skeletal Measurements by Quantitative Ultrasound in Adolescents and Young Women with Anorexia Nervosa. *J. Pediatr.* Mar 2007;150(3):286-290 e281.
121. Divasta AD, Laufer MR, Gordon CM. Bone density in adolescents treated with a GnRH agonist and add-back therapy for endometriosis. *J. Pediatr. Adolesc. Gynecol.* Oct 2007;20(5):293-297.
122. Haagenson A, Ringelheim J, Feldman HA, Gordon CM. Low prevalence of vitamin D deficiency among adolescents with anorexia nervosa. *Pediatric Academic Societies Annual Meeting.* 2007.
123. Clowes JA, Hannon RA, Yap TS, Hoyle NR, Blumsohn A, Eastell R. Effect of feeding on bone turnover markers and its impact on biological variability of measurements. *Bone.* Jun 2002;30(6):886-890.

124. Rockett HR, Wolf AM, Colditz GA. Development and reproducibility of a food frequency questionnaire to assess diets of older children and adolescents. *J. Am. Diet. Assoc.* Mar 1995;95(3):336-340.
125. Berkey CS, Rockett HR, Field AE, et al. Activity, dietary intake, and weight changes in a longitudinal study of preadolescent and adolescent boys and girls. *Pediatrics.* Apr 2000;105(4):E56.
126. Rifas-Shiman SL, Gillman MW, Field AE, et al. Comparing physical activity questionnaires for youth: seasonal vs annual format. *Am. J. Prev. Med.* May 2001;20(4):282-285.
127. Taveras EM, Rifas-Shiman SL, Field AE, Frazier AL, Colditz GA, Gillman MW. The influence of wanting to look like media figures on adolescent physical activity. *J. Adolesc. Health.* Jul 2004;35(1):41-50.
128. Blair SN, Haskell WL, Ho P, et al. Assessment of habitual physical activity by a seven-day recall in a community survey and controlled experiments. *Am. J. Epidemiol.* Nov 1985;122(5):794-804.
129. Arroll B, Jackson R, Beaglehole R. Validation of a three-month physical activity recall questionnaire with a seven-day food intake and physical activity diary. *Epidemiology.* Jul 1991;2(4):296-299.
130. Beck AT, Brown G, Steer RA. *Beck Depression Inventory II manual*. San Antonio, TX: The Psychological Corporation; 1996.
131. Spielberger CD, Gorsuch RL, Lushene R, Vagg PR, Jacobs GA. *Manual for the state-trait inventory*. Palo Alto: Consulting Psychologists Press; 1983.
132. Garner DM, Olmsted MP, Bohr Y, Garfinkel PE. The eating attitudes test: psychometric features and clinical correlates. *Psychol. Med.* Nov 1982;12(4):871-878.
133. Beck AT, Ward CH, Mendelson M, Mock J, Erbaugh J. An inventory for measuring depression. *Arch. Gen. Psychiatry.* Jun 1961;4:561-571.
134. Speilberger CD, Gorsuch RL, Lushene R, Vagg PR, Jacobs GA. Manual for the state-trait inventory. Palo Alto: Consulting Psychologists Press; 1983.
