# Supplementary material for: Low-Magnitude Mechanical Signals to Preserve Skeletal Health in Female Adolescents With Anorexia Nervosa: A Randomized Clinical Trial
Source: JAMA Netw Open. 2024 Oct 31;7(10):e2441779. doi: 10.1001/jamanetworkopen.2024.41779 (PMC11528308; doi:10.1001/jamanetworkopen.2024.41779)
Supplement: Supplement 2. — eTable 1. Demographic and clinical data for n = 21 participants with %mBMI ≤85 eTable 2. Demographic and clinical data for n = 8 severely malnourished patients with %mBMI ≤80 eTable 3. Measures of radial pQCT at baseline and after 6 months of LMMS treatment or placebo in young women with anorexia nervosa (N = 50) [file jamanetwopen-e2441779-s002.pdf]

Supplementary Online Content

DiVasta AD, Stamoulis C, Rubin CT, et al. Preservation of skeletal health in ambulatory female adolescents with anorexia nervosa: a randomized clinical trial. *JAMA Netw Open*. 2024;7(10):e2441779. doi:10.1001/jamanetworkopen.2024.41779

eTable 1. Demographic and clinical data for n = 21 participants with %mBMI ≤ 85
eTable 2. Demographic and clinical data for n = 8 severely malnourished patients with %mBMI ≤ 80
eTable 3. Measures of radial pQCT at baseline and after 6 months of LMMS treatment or placebo in young women with anorexia nervosa (N = 50)

This supplementary material has been provided by the authors to give readers additional information about their work.

© 2024 DiVasta AD et al. *JAMA Network Open.*

eTable 1. Demographic and clinical data for n = 21 participants with %mBMI ≤ 85

	**Participants with %mBMI ≤ 85 (n = 21)**	
	Median (IQR)	Min, Max
**Age**, y	17.1 (5.2)	12.6, 26.1
**Height**, cm	161.3 (6.1)	154.9, 174.5
**Weight**, kg	43.3 (5.6)	36.8, 51.9
**BMI**, kg/m^2	16.4 (1.3)	14.6, 18.2
**% of median BMI for age**	80.2 (5.1)	68.5, 84.8
**Duration of AN**, mos	6.0 (39.5)	0, 120.0
**Duration of amenorrhea**, mos	5.5 (4.0)	0, 78.0
**Age at menarche**, y	12.0 (2.0)	10.0, 14.0
**25(OH)D**, ng/mL	37.5 (16.2)	18.3, 68.1
**PTH**, pg/mL	17.0 (8.8)	8.0, 36.0
**DXA MEASURES**		
**Total body Z-score**	-0.6 (1.3)	-3.3, 1.4
**Spine Z-score**	-1.0 (1.1)	-2.8, 0.2
**Total hip Z-score**	-0.7 (1.5)	-3.1, 1.8
	**N (%)**	
**DXA Total body:**		
**Z ≤ –1**	9 (42.9)	
**Z ≤ –2**	2 (9.5)	
**DXA Spine:**		
**Z ≤ –1**	11 (52.4)	
**Z ≤ –2**	2 (9.5)	
**DXA Total hip:**		
**Z ≤ –1**	10 (47.6)	
**Z ≤ –2**	2 (9.5)	

%mBMI, % of median body mass index for age; LMMS, low-magnitude mechanical stimulation; IQR, interquartile range; BMI, body mass index; AN, anorexia nervosa; 25(OH)D, 25-hydroxy-vitamin D; PTH, parathyroid hormone; DXA, dual energy X-ray absorptiometry

© 2024 DiVasta AD et al. *JAMA Network Open.*

eTable 2. Demographic and clinical data for n = 8 severely malnourished patients with %mBMI ≤ 80

	**Participants with %mBMI ≤ 80 (n = 8)**	
	Median (IQR)	Min, Max
**Age**, y	16.9 (2.3)	14.2, 23.5
**Height**, cm	162.5 (8.8)	154.9, 174.5
**Weight**, kg	40.2 (4.3)	36.8, 49.6
**BMI**, kg/m^2	16.1 (1.3)	14.6, 16.6
**% of median BMI for age**	77.5 (5.9)	68.5, 79.1
**Duration of AN**, mo	2.5 (21.5)	0, 88.0
**Duration of amenorrhea**, mo	6.0 (4.0)	1.0, 78.0
**Age at menarche**, y	13.0 (1.8)	11.0, 14.0
**25(OH)D**, ng/mL	31.8 (14.2)	18.3, 47.1
**PTH**, pg/mL	17.0 (9.6)	8.0, 36.0
**DXA MEASURES**		
**Total body Z-score**	-0.5 (1.5)	-3.3, 1.4
**Spine Z-score**	-1.1 (1.2)	-2.8, 0.2
**Total hip Z-score**	-0.2 (1.3)	-3.1, 1.8
	**N (%)**	
**DXA Total body:**<br>**Z ≤ –1**<br>**Z ≤ –2**	 3 (37.5)<br>1 (12.5)	
**DXA Spine:**<br>**Z ≤ –1**<br>**Z ≤ –2**	 5 (62.5)<br>1 (12.5)	
**DXA Total hip:**<br>**Z ≤ –1**<br>**Z ≤ –2**	 2 (25.0)<br>1 (12.5)	

%mBMI, % of median body mass index for age; LMMS, low-magnitude mechanical stimulation; IQR, interquartile range; BMI, body mass index; AN, anorexia nervosa; 25(OH)D, 25-hydroxy-vitamin D; PTH, parathyroid hormone; DXA, dual energy X-ray absorptiometry

© 2024 DiVasta AD et al. *JAMA Network Open.*

eTable 3. Measures of radial pQCT at baseline and after 6 months of LMMS treatment or placebo in young women with anorexia nervosa (N = 50)

**Radius 4% site (non-weight-bearing trabecular bone)**				
Total bone vBMD (mg/cm^3)				
LMMS	321.1 (67.4)	332.8 (34.8)	0.32	0.26
Placebo	346.6 (46.2)	337.8 (22.5)	0.53	
Trabecular vBMD (mg/cm^3)				
LMMS	215.7 (37.8)	218.6 (19.4)	0.90	0.40
Placebo	211.9 (38.7)	221.4 (14.3)	0.50	
Total bone CSA (mm^2)				
LMMS	253.4 (68.3)	257.6 (32.3)	0.85	0.88
Placebo	258.1 (70.5)	260.9 (21.9)	0.80	
Trabecular CSA (mm^2)				
LMMS	192.3 (58.2)	180.5 (41.6)	0.29	0.29
Placebo	176.2 (74.5)	181.0 (54.5)	0.54	
Cortical vBMD (g/cm^3)				
LMMS	583.0 (78.2)	600.2 (89.3)	0.60	0.13
Placebo	631.9 (90.4)	601.1 (71.3)	0.19	
**Radius 66% site (non-weight-bearing cortical bone)**				
Total bone vBMD (mg/cm^3)				
LMMS	731.5 (108.1)	707.5 (28.8)	0.20	0.12
Placebo	711.7 (135.4)	710.1 (25.8)	0.57	
Total bone CSA (mm^2)				
LMMS	109.3 (28.7)	111.0 (10.6)	0.42	0.68
Placebo	114.2 (35.0)	114.3 (10.5)	0.60	
Cortical vBMD (g/cm^3)				
LMMS	1093.4 (50.7)	1096.3 (38.4)	0.37	0.20
Placebo	1080.4 (74.0)	1082.3 (31.9)	0.78	
Cortical CSA (mm^2)				
LMMS	66.7	64.9 (6.7)	0.97	0.91
Placebo	62.8	66.4 (5.9)	0.59	

© 2024 DiVasta AD et al. *JAMA Network Open.*

Cortical thickness (mm)				
LMMS	2.18 (0.4)	2.16 (0.2)	0.52	0.48
Placebo	2.11 (0.6)	2.15 (0.2)	0.50	
Cortical section modulus (mm^3)				
LMMS	309.6 (81.2)	314.2 (53.7)	0.19	0.27
Placebo	323.7 (143.2)	316.9 (37.5)	0.82	
Max. muscle CSA (mm^2)				
LMMS	2063.0 (340.2)	2204.2 (207.1)	0.21	0.96
Placebo	2113.8 (446.5)	2245.6 (172.0)	0.32	
Strength-Strain Index (mm^3)				
LMMS	251.3 (78.3)	253.8 (48.4)	0.49	0.47
Placebo	252.7 (78.8)	256.1 (36.1)	0.89	

* Estimates adjusted for patient age, duration of AN, and weight gain
LMMS, low-magnitude mechanical stimulation; vBMD, volumetric bone mineral density; CSA, cross-sectional area

© 2024 DiVasta AD et al. *JAMA Network Open.*
