# Supplementary material for: Low-Magnitude Mechanical Signals to Preserve Skeletal Health in Female Adolescents With Anorexia Nervosa: A Randomized Clinical Trial
Source: JAMA Netw Open. 2024 Oct 31;7(10):e2441779. doi: 10.1001/jamanetworkopen.2024.41779 (PMC11528308; doi:10.1001/jamanetworkopen.2024.41779)
Supplement: Supplement 3. — Data Sharing Statement [file jamanetwopen-e2441779-s003.pdf]

# Data Sharing Statement

DiVasta. Preservation of Skeletal Health in Ambulatory Female Adolescents With Anorexia Nervosa. *JAMA Netw Open*. Published October 31, 2024.
doi:10.1001/jamanetworkopen.2024.41779

**Data**
**Additional Information:** clinicaltrials.gov NCT01100567
**Data available:** Yes
**Data types:** Deidentified participant data
**How to access data:** amy.divasta@childrens.harvard.edu
**When available:** With publication

**Supporting Documents**
**Document types:** None

**Additional Information**
**Who can access the data:** researchers whose proposed use of the data has been approved
**Types of analyses:** For questions related to evidence based care of patients with anorexia nervosa
**Mechanisms of data availability:** After approval of a proposal and a signed data access agreement across institutions
